# *Francisella tularensis* Harvests Nutrients Derived via ATG5-Independent Autophagy to Support Intracellular Growth

**DOI:** 10.1371/journal.ppat.1003562

**Published:** 2013-08-15

**Authors:** Shaun Steele, Jason Brunton, Benjamin Ziehr, Sharon Taft-Benz, Nathaniel Moorman, Thomas Kawula

**Affiliations:** Department of Microbiology and Immunology, University of North Carolina at Chapel Hill, Chapel Hill, North Carolina, United States of America; Institut Pasteur, France

## Abstract

*Francisella tularensis* is a highly virulent intracellular pathogen that invades and replicates within numerous host cell types including macrophages, hepatocytes and pneumocytes. By 24 hours post invasion, *F. tularensis* replicates up to 1000-fold in the cytoplasm of infected cells. To achieve such rapid intracellular proliferation, *F. tularensis* must scavenge large quantities of essential carbon and energy sources from the host cell while evading anti-microbial immune responses. We found that macroautophagy, a eukaryotic cell process that primarily degrades host cell proteins and organelles as well as intracellular pathogens, was induced in *F. tularensis* infected cells. *F. tularensis* not only survived macroautophagy, but optimal intracellular bacterial growth was found to require macroautophagy. Intracellular growth upon macroautophagy inhibition was rescued by supplying excess nonessential amino acids or pyruvate, demonstrating that autophagy derived nutrients provide carbon and energy sources that support *F. tularensis* proliferation. Furthermore, *F. tularensis* did not require canonical, ATG5-dependent autophagy pathway induction but instead induced an ATG5-independent autophagy pathway. ATG5-independent autophagy induction caused the degradation of cellular constituents resulting in the release of nutrients that the bacteria harvested to support bacterial replication. Canonical macroautophagy limits the growth of several different bacterial species. However, our data demonstrate that ATG5-independent macroautophagy may be beneficial to some cytoplasmic bacteria by supplying nutrients to support bacterial growth.

## Author Summary

*Francisella tularensis* is a highly virulent bacterial pathogen that infects hundreds of different animal species including humans. During infection, *F. tularensis* bacteria invade and rapidly multiply inside host cells. Within the host cell environment, basic nutrients that bacteria require for growth are in limited supply, and the majority of nutrients are tied up in complex molecules that are not readily available in forms that can be used by bacteria. In this study we asked and answered a very simple question; how does *F. tularensis* harvest sufficient carbon and energy sources from the host cell to support rapid intracellular growth? We found that *F. tularensis* induces a host recycling pathway in infected cells. Thus the host cell degrades nonessential proteins and releases amino acids. *F. tularensis* harvests the host-derived amino acids to generate energy and build its own more complex molecules. When we inhibited the host recycling pathway, growth of the intracellular bacteria was limited. Therefore, manipulation of host cell metabolism may be a means by which we can control the growth of intracellular bacterial pathogens during infection.

## Introduction

When intracellular bacterial pathogens invade host cells, the bacteria must scavenge energy sources and anabolic substrates from the nutrient-limited intracellular environment. Most of the potential nutrient sources inside a host cell are stored within complex structures such as lipid droplets, glycogen and proteins, which are not immediately available to intracellular pathogens. To obtain nutrients for proliferation, intracellular bacteria must degrade these complex structures into their constituents (fatty acids, carbohydrates and amino acids respectively) or increase nutrient import. The strategies that bacteria use to acquire nutrients could potentially have widespread effects on the host cell. For example, pathogens that import amino acids from the host cell cytoplasm may starve the cell. Host cell amino acid starvation leads to mammalian target of rapamycin (mTOR) inhibition, thereby inhibiting mRNA transcription and other critical cellular homeostatic processes [1].Thus, nutrient acquisition is an important step in the pathogenesis of intracellular bacteria and is critical to understand how a pathogen interacts with the host.

Autophagy is a highly conserved eukaryotic cell process that can be initiated by a variety of factors such as amino acid starvation, energy depletion, mTOR inhibition and immune signaling [2], [3]. Autophagy is a process by which multi-membranous vesicles called autophagosomes surround and degrade cellular constituents (during starvation) or cytoplasmic bacteria (during infection through a related innate immune response termed xenophagy [4]). The autophagosomes fuse with lysosomes to become autolysosomes, which then degrade the engulfed material. During starvation, autophagy can degrade nonessential proteins, thereby releasing free amino acids that are recycled into new proteins. Current studies of the interactions between host autophagy and intracellular bacterial pathogens are primarily focused on xenophagy [5]–[7]. However, a few intracellular pathogens are known to benefit from autophagy [8]–[10]. Autophagosome formation is induced during infection with *Anaplasma phagocytophilum* and the autophagy derived nutrients are harvested and used by *A. phagocytophilum* to enhance intracellular replication [9]. Likewise, dengue virus uses autophagic byproducts to acquire lipids for viral replication [10]. Pathogens such as *Listeria monocytogenes* express active mechanisms that prevent bacterial degradation via xenophagy, yet autophagy still occurs in the infected cell and has the potential to provide nutrient sources for the bacteria [11]. These and other recent studies highlight the potential role of autophagy in providing nutrients or other benefits for intracellular pathogens.

*Francisella tularensis* is a facultative intracellular bacterium that infects over 200 different species (from amoeba to humans) [12]. The highly virulent *F. tularensis* subsp. *tularensis* Schu S4 strain has an infectious dose of fewer than 25 bacteria and a mortality rate of 30–60% in untreated pneumonic infections [13], [14]. *F. tularensis* infects a diverse range of cell types including macrophages, which are a key replicative niche for *F. tularensis* in humans and other susceptible mammals. *F. tularensis* also invades and replicates within several other cell types including epithelial cells and endothelial cells [12], [15]. *F. tularensis* enters the host cell through phagocytosis and proceeds to escape the phagosome and replicate in the host cell cytoplasm. By 24 hours post inoculation, *F. tularensis* replicates up to 1000-fold inside host cells. This rapid intracellular replication plays a major role in *F. tularensis* pathogenesis but the mechanisms by which this organism acquires nutrients are not well characterized. Therefore, we sought to determine how these nutrients become available to support efficient *F. tularensis* intracellular replication.

In primary murine macrophages, *F. tularensis* induces the formation of a multi-membranous, autophagosome-like structure termed the Francisella containing vacuole (FCV) through an autophagy related process [16]. FCV formation occurs between 20 and 36 hours post inoculation, after the majority of *F. tularensis* replication has taken place. Blocking FCV formation late during infection does not increase *F. tularensis* proliferation, suggesting that FCV formation does not play a role in controlling intracellular *F. tularensis* replication [16]. However, the formation of FCVs hints that autophagy may be induced during *F. tularensis* infection. Additionally, replication deficient and chloramphenicol treated *F. tularensis* bacteria, but not wild type *F. tularensis* bacteria, are degraded via canonical autophagy [17]. This observation implies that *F. tularensis* avoids xenophagy. Lastly, treating *F. tularensis* infected macrophages 2 hours post inoculation with chloroquine or autophagy- inhibiting levels of ammonium chloride impairs *F. tularensis* intracellular replication [18]–[20]. Although chloroquine and ammonium chloride inhibit acidification of cellular compartments and have broad effects on the host cell, these data raise the intriguing possibility that autophagy may contribute to *F. tularensis* intracellular replication.

Taken together these observations suggest that intracellular *F. tularensis* avoids xenophagy yet induces autophagy or an autophagy-like process that contributes to *F. tularensis* proliferation. We therefore examined the potential role of autophagy in aiding *F. tularensis* intracellular growth.

## Results

### Host cell constituents are sufficient to support *F. tularensis* intracellular proliferation

**Figure 1:**
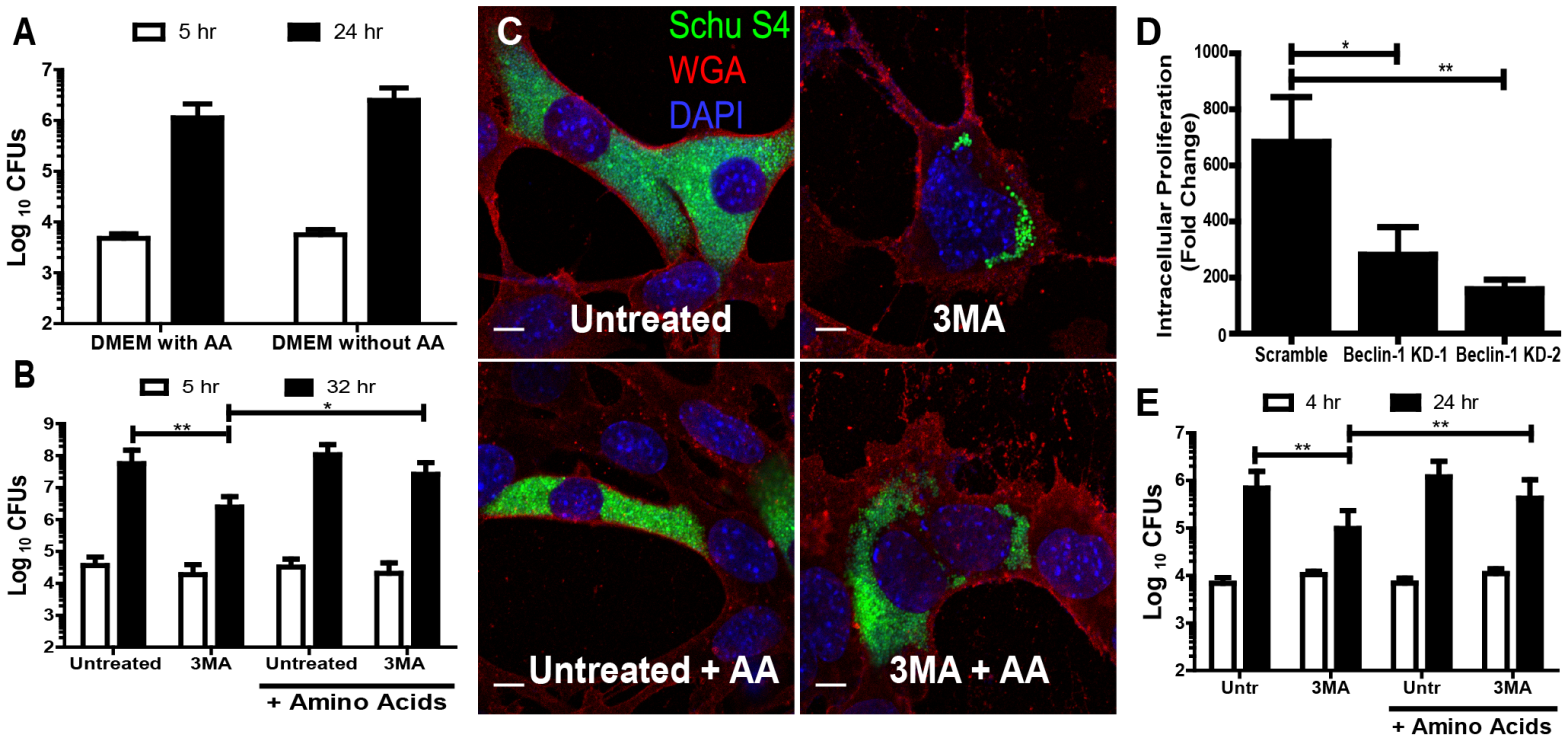
Autophagy derived nutrients enhance *F. tularensis* intracellular growth. (**A**) Number of intracellular *F. tularensis* 5 and 24 hours post-inoculation of MEFs cultured in DMEM with or without amino acids (mean +/− SD, 4 independent experiments). (**B**) Number of intracellular *F. tularensis* 5 and 32 hours post-inoculation of untreated and 3MA treated MEFs with or without amino acid supplementation (AA) (mean +/− SD, 3 independent experiments). (**C**) Representative confocal microscopy images of infected MEFs 32 hours post inoculation that were untreated, 3MA treated or each treatment with amino acid supplementation. Each scale bar represents 10 µm. GFP- Schu S4 bacteria are depicted in green, DAPI (nucleus) in blue, and wheat germ agglutinin (WGA) (plasma membrane) in red. (**D**) Intracellular bacterial proliferation from 5 to 24 hours post-inoculation of MEFs transduced with a scrambled control or one of two different shRNA's to Beclin-1 (mean +/− SEM, 5 independent experiments). (**E**) The number of intracellular *F. tularensis* 4 and 24 hours post inoculation of untreated or 3MA treated hMDMs with or without amino acid supplementation (AA) (mean +/− SD, 4 independent experiments). (* p<0.05, **p<0.01).

*F. tularensis* replicates efficiently and rapidly in host cells. Indeed, transmission electron microscopy analysis showed that *F. tularensis* consumed over half of the area of the cell cytoplasm of infected mouse embryonic fibroblasts (MEFs) by 16 hours post inoculation (Figure S1). *F. tularensis* cannot make all of the nutrients it needs *de novo* and must interact with the host to acquire certain metabolites to support rapid proliferation. In particular, *F. tularensis* is auxotrophic for 13 amino acids, some of which mammalian cells also do not synthesize. Thus, for sustained proliferation within infected cells, the bacteria must either take up amino acids imported by the host cell or degrade host proteins and reuse the resulting amino acids. To distinguish between these possibilities, we determined if decreasing the availability of free amino acids limited *F. tularensis* intracellular growth. We replaced the media on infected MEFs with media lacking amino acids at 3 hours post inoculation. *F. tularensis* replicated to similar numbers with or without amino acids present in the tissue culture media (Figure 1A). This result demonstrates that *F. tularensis* can acquire the amino acids it needs to sustain growth directly from the host cell. Since the majority of host amino acids are typically sequestered in proteins inside the cell, protein degradation likely occurs to provide sufficient amino acids to support *F. tularensis* intracellular growth. Additionally, amino acid depletion results in starvation induced autophagy [21]. Starvation induced autophagy will degrade proteins to produce amino acids. Thus, *F. tularensis* may take advantage of host cell autophagy to acquire free amino acids.

### Autophagy supplies energy and anabolic substrates that support *F. tularensis* growth in fibroblasts

To determine if autophagy had any impact on *F. tularensis* intracellular growth we measured bacterial replication inside cells treated with several different autophagy inhibitors. MEFs were treated with 3-methyladenine (3MA), which inhibits autophagosome formation, thereby blocking autophagy. *F. tularensis* replication inside 3MA treated MEFs was significantly reduced (Figure 1B), suggesting that intracellular *F. tularensis* benefit from host cell autophagy. Since autophagy is both a starvation response and a process by which damaged organelles and non-essential proteins are degraded we considered the possibility that *F. tularensis* may scavenge and utilize amino acids released by this process. We therefore wanted to determine if exogenous amino acid supplementation would rescue *F. tularensis* growth in MEFs that have impaired autophagy function. Indeed, *F. tularensis* intracellular growth in the presence of 3MA was restored by the addition of excess amino acids to the culture media (Figure 1B). These results, which were corroborated using confocal fluorescence microscopy of cells infected with GFP-expressing *F. tularensis* Schu S4 (Figure 1C) indicate that autophagy provides a source of nutrients that support *F. tularensis* replication.

To determine if degradative autophagy was responsible for optimal bacterial growth, we quantified *F. tularensis* intracellular growth in the presence of Bafimoycin A(1) (Baf) or chloroquine (CQ), each of which inhibits autophagy by blocking functional autolysosome formation. We tested the effect of these drugs on *F. tularensis* replication kinetics by infecting MEFs with *F. tularensis* containing a bioluminescence reporter plasmid (Schu S4-LUX) [22] and measuring luminescence every 30 minutes to determine the bacterial growth kinetics. The limit of detection for this assay was approximately 50 relative light units (RLUs) or approximately 10^5^ bacteria in a 96 well format (data not shown). We verified this technique by treating *F. tularensis* infected cells with 3MA or 3MA supplemented with amino acids and observed similar results to the standard intracellular proliferation assays (Figure S2A, S2B). Additionally, CQ significantly reduced *F. tularensis* growth and amino acid supplementation rescued bacterial growth in CQ treated cells (Figure S2C, S2D). Similar to 3MA and CQ, treating MEFs with Baf also significantly reduced *F. tularensis* intracellular growth and growth was rescued with amino acid supplementation (Figure S2E, S2F). None of the inhibitors affected *F. tularensis* growth in broth culture (Figure S3B). Although 3MA, CQ, and Baf were each cytotoxic to MEFs, viability was comparable between treatments with and without amino acid supplementation (Figure S3A). Thus, the observed rescue was not due to increased eukaryotic cell viability upon amino acid supplementation.

Since all chemical inhibitors have the potential to confer off-target or non-specific effects on host cell processes we wanted to confirm the inhibitor results using genetic approaches. Beclin-1 is required for autophagosome formation in most autophagy pathways [23]. We therefore reasoned that depletion of Beclin-1 should limit bacterial replication if autophagy is required for *F. tularensis* growth. We created two Beclin-1 knock down MEF cell lines, Beclin-1 KD-1 and KD-2 that expressed 63.8% (+/−14.4%) and 59.2% (+/−12.9%) of the scrambled shRNA control Beclin-1 mRNA, respectively (Figure S4). Despite the relatively modest reduction of Beclin-1 mRNA *F. tularensis* replication was significantly reduced in the knockdown cell lines compared to the scrambled control (Figure 1D); supporting the conclusion that autophagy may have a pro-bacterial role in *F. tularensis* infected cells. Interestingly, the infection frequency of the knock down cell lines was approximately 2-fold higher than the scrambled control (data not shown) suggesting that Beclin-1 activity may modestly impair *F. tularensis* infection of host cells.

### Autophagy supports *F. tularensis* replication in primary human monocyte derived macrophages

During the course of infection *F. tularensis* invade and replicate within many different cell lineages and types. Intracellular growth properties of *F. tularensis* vary depending on host cell type. For example, *F. tularensis* infects monocytes at a significantly higher frequency than epithelial cells or fibroblasts. On the other hand, *F. tularensis* intracellular growth is more prolonged, and achieves nearly 10-fold higher peak numbers in MEFs as compared to monocytes (data not shown). Growth within monocytes is a property that is fundamental to *F. tularensis* virulence. *F. tularensis* is also a human pathogen; we therefore wanted to determine the relevance of autophagy in supporting *F. tularensis* growth within human macrophages. Inhibition of autophagy with 3MA significantly decreased *F. tularensis* growth in hMDMs, and growth was rescued in 3MA treated hMDMs by supplementing the media with excess amino acids (Figure 1E). Therefore, autophagy provides amino acids that support *F. tularensis* intracellular growth in primary human monocytes, a property that is crucial to *F. tularensis* pathogenesis.

### *F. tularensis* infection increases autophagic flux

**Figure 2:**
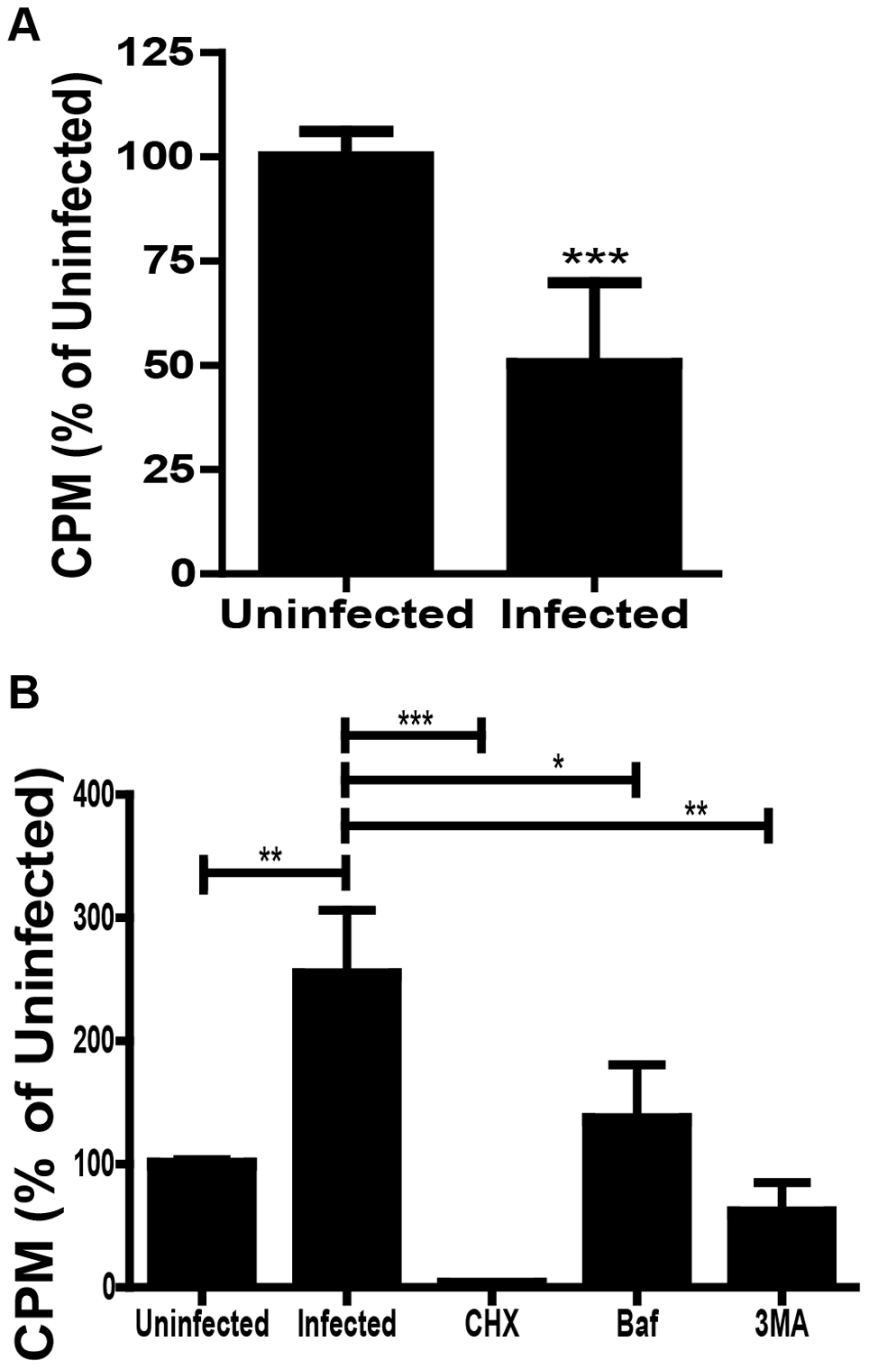
Autophagic flux and transfer of amino acids in *F. tularensis* infected cells. (**A**) S^35^ counts in the TCA insoluble fraction of uninfected or infected J774 cells 16 hours post inoculation (mean +/− SEM, 3 independent experiments). (**B**) S^35^ counts in the bead purified *F. tularensis* fraction that was TCA insoluble from either uninfected MEFs or *F. tularensis* infected MEFs exposed to the indicated treatments (mean +/− SEM, 6 independent experiments) (* p<0.05, **p<0.01, *** <0.001).

We compared the rate of degradation of long-lived proteins in uninfected and infected cells to determine if *F. tularensis* infection impacted autophagic flux. Since we were attempting to quantify a specific infected host cell response we performed this analysis in the J774A.1 monocyte cell line (J774) where the *F. tularensis* infection frequency is much greater than the infection frequency in MEFs (data not shown). We first labeled cellular proteins by incubating J774 cells in media containing ^35^S methionine and cysteine for 18 hours and chased for 2 hours to remove any remaining labeled free amino acids. The labeled cells were inoculated with *F. tularensis* and incubated for 16 hours. Following infection, infected cells had a 49.5%+/−7.9% (Average +/− SEM) decrease of ^35^S label in the TCA insoluble fraction of the cytoplasm (which will primarily contain proteins) compared to uninfected J774 cells (Figure 2A). Thus, infected cells had increased turnover of long lived proteins than uninfected cells. This result is consistent with autophagy induction in *F. tularensis* infected J774 cells. The decrease of total ^35^S label in both host and bacterial proteins in infected cells may indicate that the transfer of amino acids from the host to the bacteria is inefficient or that the majority of amino acids are used by *F. tularensis* for energy rather than protein synthesis. Uninfected and infected J774 cells had similar levels of cytotoxicity at 16 hours post inoculation, indicating that the loss of label in infected compared to uninfected cells was not due to cell lysis (Figure S3D).

### Autophagy derived amino acids are transferred from host proteins to *F. tularensis*

To confirm that *F. tularensis* imports amino acids derived from host proteins, we monitored transfer of radiolabelled amino acids from host proteins into bacterial proteins. MEFs were first metabolically labeled with ^35^S-labeled methionine and cysteine for 18 hours to fully label all host proteins. Then the radiolabel was removed and the cells were incubated in unlabeled media for two hours prior to infection with *F. tularensis* to remove ^35^S that was not incorporated into protein. At 16 hours post infection (18 hours after the radiolabel was removed) we lysed the MEFs and purified *F. tularensis* by mixing cell lysate from either uninfected or infected cells with magnetic beads linked to an anti- *F. tularensis* lipopolysaccharide (LPS) antibody. We then determined if *F. tularensis* proteins contained radiolabeled amino acids by examining the trichloroacetic acid (TCA) insoluble fraction of purified *F. tularensis*. There was a significant increase of radiolabel in the TCA insoluble, *F. tularensis* bead purified fraction from infected MEFs as compared to uninfected control samples (Figure 2B). Indeed, 6.22%+/−4.15% (average +/− SEM, n = 5 samples) of the TCA insoluble radiolabel present prior to infection transferred to the bacteria during the 16 hour infection. To control for possible direct transfer of labeled amino acids that were not incorporated into host proteins we analyzed infected MEFs that were treated with cycloheximide during ^35^S labeling prior to infection. There were negligible amounts of radiolabel present in the bead purified fraction of cycloheximide treated cells (Figure 2B). *F. tularensis* survived and replicated within cycloheximide pre-treated cells and *F. tularensis* was present in the bacterial purified fraction (data not shown). Thus, host cell lysis due to the cycloheximide treatment was not solely responsible for the lack of radiolabel in the bacterial fraction. ^35^S radiolabel was primarily incorporated into host proteins, rather than as free ^35^S labeled amino acids. Taken together, these data demonstrate that *F. tularensis* synthesized proteins using amino acids derived from host cell proteins.

Treating the radiolabeled cells with either Baf or 3-MA resulted in significantly decreased incorporation of the radiolabel by *F. tularensis* (Figure 2C). Since *F. tularensis* proliferation is reduced in 3MA and Baf treated MEFs, several fold fewer bacteria were present in the bacteria purified fraction of the treated MEFs (data not shown). Nevertheless, the median ^35^S counts per bacteria were significantly lower in the 3MA or Baf treated samples compared to untreated samples (untreated: 0.016 CPM/bacteria, 3MA: 0.000 CPM/bacteria, Baf: 0.000 CPM/bacteria [n = 3 experiments done in duplicate]). Therefore, transfer of radiolabeled amino acids to bacterial proteins was reduced by both 3MA and Baf treatment, indicating that under normal culture conditions, amino acids derived by the degradation of host cell proteins via autophagy were used by *F. tularensis*.

### *F. tularensis* uses autophagy by-products primarily for energy

**Figure 3:**
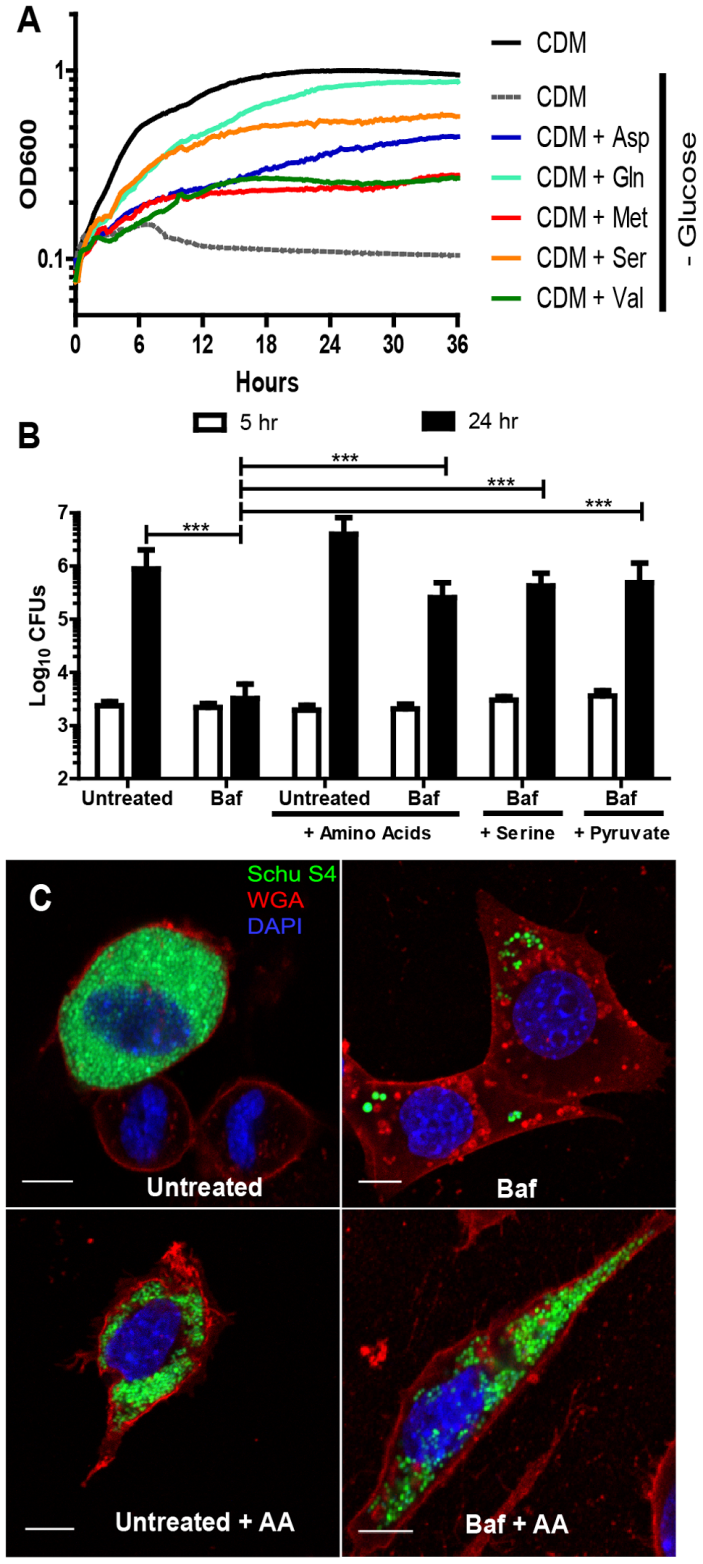
*F. tularensis* uses autophagy derived nutrients for energy and anabolic substrates. (**A**) *F. tularensis* growth in Chamberlin's defined media (CDM) and CDM lacking glucose but supplemented with 30 mM of a specific amino acid or other carbon source (each point represents an average of triplicate wells, 3 independent experiments). (**B**) Number of intracellular *F tularensis* 5 and 24 hours post-inoculation of untreated or Baf treated MEFs. MEFs were supplemented with a 12 mM amino acid mixture, 15 mM serine, or 18 mM pyruvate (mean +/− SD, 3 independent experiments). (**C**) Representative confocal microscopy images of infected MEFs 24 hours post inoculation that were untreated, Baf treated or each condition plus amino acid supplementation. Each scale bar represents 10 µm. GFP- Schu S4 bacteria are depicted in green, nuclei (DAPI) in blue, and wheat germ agglutinin (WGA) (plasma membrane) in red. (***p<0.001).

*F. tularensis* is capable of using amino acids as an energy source when simple carbohydrates such as glucose are not available (Figure 3A). Thus, autophagy derived amino acids could conceivably be used by intracellular *F. tularensis* for either the synthesis of new proteins or to provide energy for other bacterial processes. Although we found that *F. tularensis* uses host-derived amino acids for protein synthesis (Figure 2B), the proportion of amino acids used for protein synthesis as opposed to energy is unknown. To determine if *F. tularensis* uses autophagy-derived amino acids primarily as anabolic precursors or as an energy source, we supplemented autophagy inhibited, *F. tularensis* infected MEFs with either serine or the metabolite pyruvate. Annotation of the *F. tularensis* genome indicates that *F. tularensis* encodes the protein L-serine dehydratase, which degrades serine directly into pyruvate. The addition of either pyruvate or serine alone rescued *F. tularensis* intracellular growth in Baf treated cells (Figure 3B). Fibroblasts cannot convert serine or pyruvate into all of 13 of the amino acids required to fulfill *F. tularensis* auxotrophies. Thus, host autophagy-derived nutrients are used by *F. tularensis* primarily as a source of energy. Although *F. tularensis* can incorporate autophagy derived amino acids into bacterial proteins (Figure 2B), these data indicate that energy, rather than amino acids for protein synthesis, was the limiting factor for *F. tularensis* proliferation in autophagy-deficient cells cultured in tissue culture media.

### ATG5 is not required for autophagy in *Francisella* infected cells

**Figure 4:**
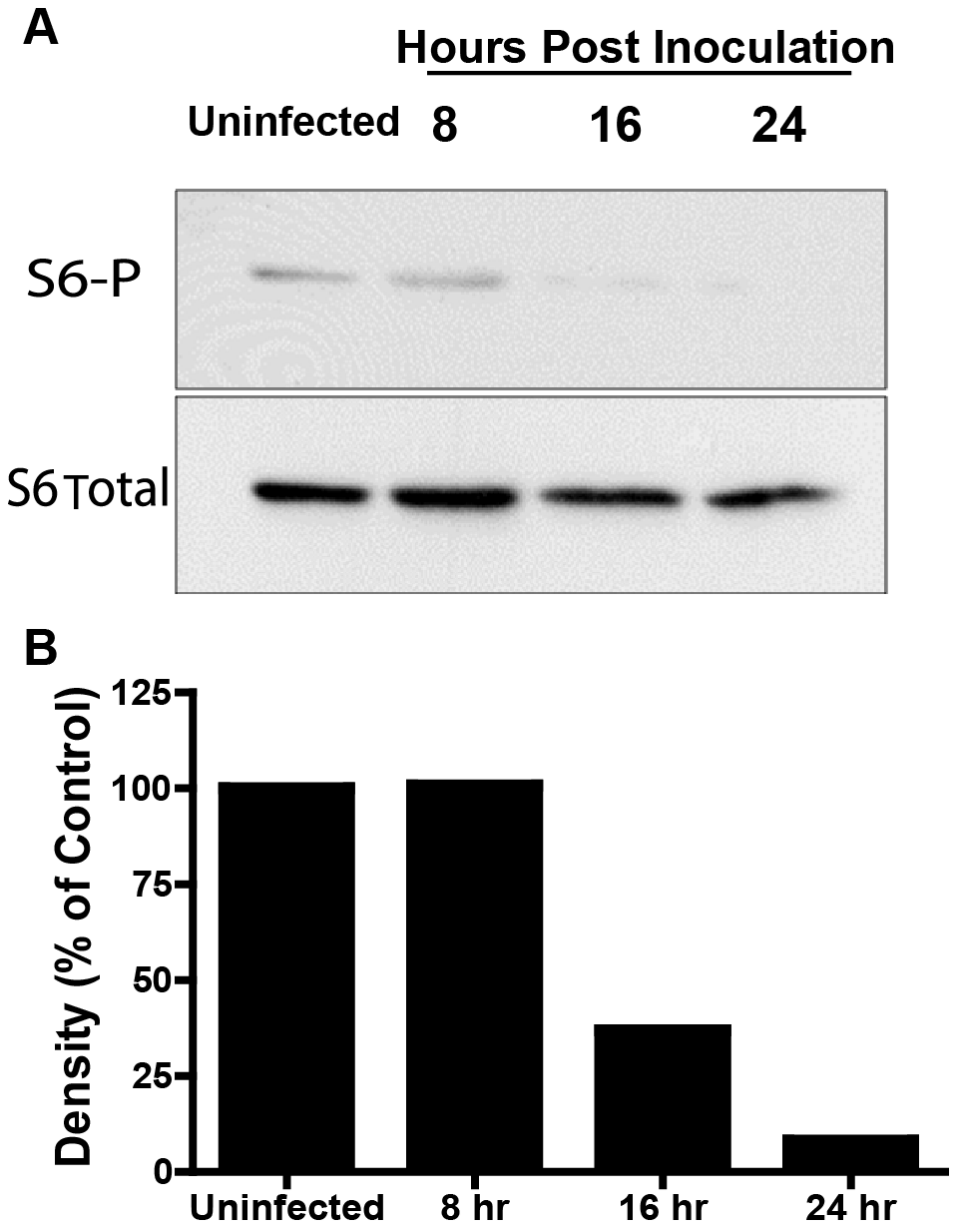
S6-P is reduced in *F. tularensis* infected cells. (**A**) A representative immunoblot of S6 ribosomal protein phosphorylation states from J774 cells uninfected and over the course of infection (3 independent experiments). (**B**) The ratio of phosphorylated ribosomal S6 to total ribosomal S6 as determined by densitometry from panel B. Densities were normalized to total ribosomal S6 protein using ImageJ and expressed as a percentage of the uninfected control.

Canonical autophagy is typically induced by the inhibition of mammalian target of rapamycin (mTOR). Thus, monitoring mTOR activity through downstream substrates such as S6 kinase is likely to correlate well with canonical autophagy induction. To determine if *F. tularensis* infection activates the autophagy signaling cascade, we assessed mTOR activity in infected J774 cells by measuring phosphorylation of the mTOR substrate S6 ribosomal protein. The ratio of phospho- S6 ribosomal protein to unphosporylated S6 ribosomal protein decreased progressively over the course of infection, which is consistent with mTOR inhibition and thus autophagy induction (Figure 4A, 4B) [24]. However, loss of phospho - S6 ribosomal protein was not evident before 8 hours post inoculation suggesting that mTOR inhibition occurred after some bacterial replication had already taken place.

**Figure 5:**
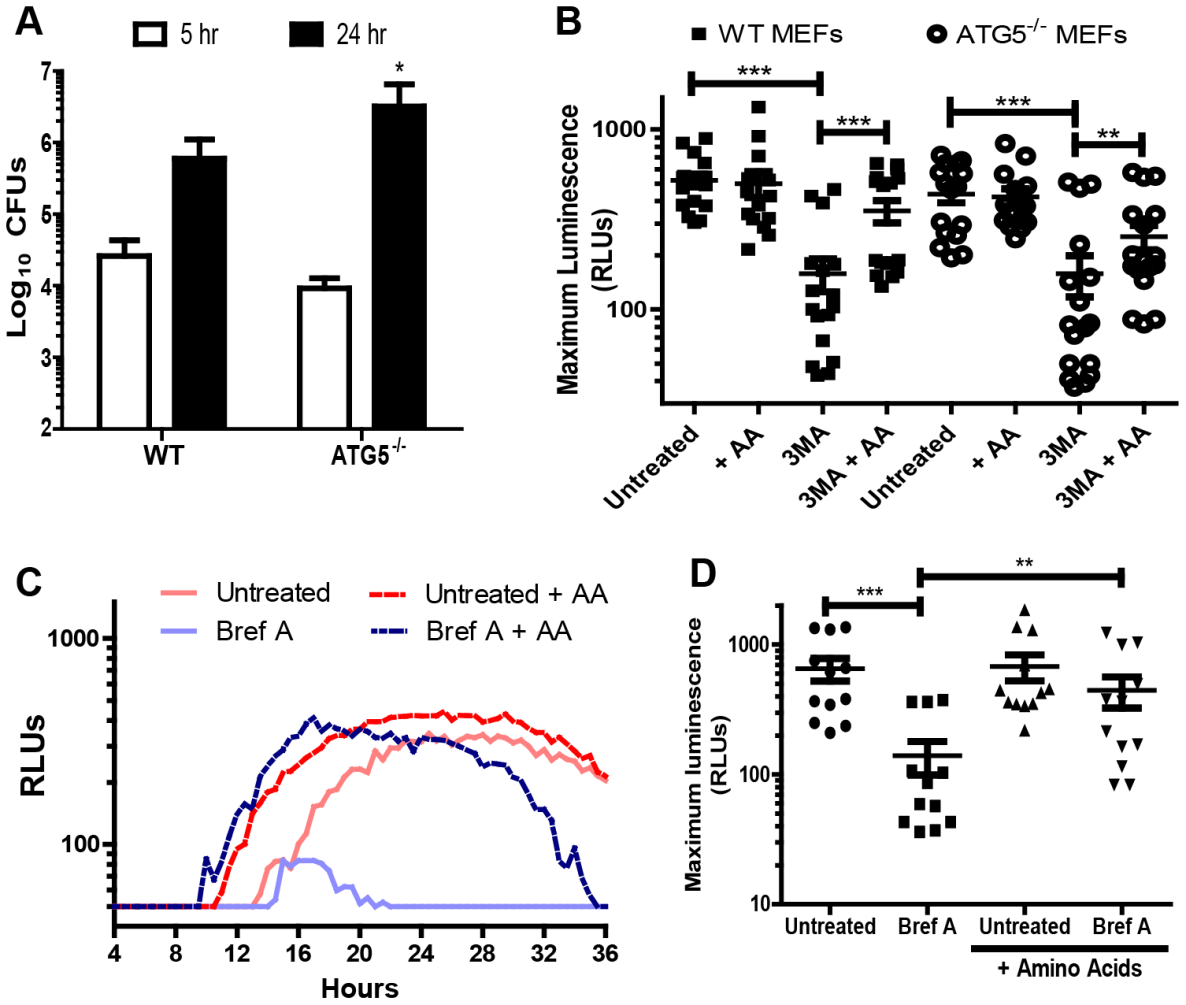
ATG5 is not required for efficient *F. tularensis* intracellular replication. (**A**) Number of intracellular *F. tularensis* 5 and 24 hours post inoculation of wild type and ATG5^−/−^ MEFs (mean +/− SD, 3 independent experiments). (**B**) Maximum luminescence values expressed in relative light units (RLUs) from kinetic growth assays for Schu S4 –LUX infected wild type or ATG5^−/−^ MEFs treated with 3MA and supplemented with amino acids as indicated (mean +/− SEM, 6 independent experiments). (**C**) Representative intracellular bacterial growth kinetics of *F. tularensis* Schu S4 LUX in untreated and brefeldin A treated J774 cells with or without amino acid supplementation (each point represents an average of triplicate wells) as measured by luminescence (3 independent experiments). (**D**) Maximum luminescence values expressed in relative light units (RLUs) from kinetic growth assays for Schu S4 LUX infected J774 cells untreated and treated with brefeldin A (4 independent experiments). (* p<0.05, ** p<.01, *** p<0.001).

In the canonical autophagy pathway the protein ATG5 is essential for autophagosome formation. Thus, we would predict that ATG5 expression would be required for autophagic degradation of host proteins to amino acids that support *F. tularensis* intracellular growth. However, it was recently shown that *F. tularensis* replicates efficiently within ATG5^−/−^ macrophages [17]. We also found that *F. tularensis* replication was not impaired in ATG5^−/−^ MEFs (Figure 5A). In fact, there was a slight but statistically significant increase in bacterial replication in ATG5^−/−^ MEFs compared to wild type MEFs (Figure 5A). Therefore, ATG5 is not required for efficient *F. tularensis* intracellular proliferation. Treatment of ATG5^−/−^ MEFs with 3MA resulted in decreased bacterial proliferation and bacterial growth was rescued by supplementing treated cells with amino acids (Figure 5B). Taken together, these data suggest that *F. tularensis* intracellular growth is supported by nutrients generated by an ATG5-independent autophagy pathway.

Unlike canonical autophagy, ATG5-independent autophagy generates autophagosomes from the trans-Golgi apparatus [25]. Brefeldin A (Bref A) inhibits ATG5-independent autophagosome formation but does not affect canonical autophagosome formation [24]. To determine if ATG5-independent autophagy provides metabolites for *F. tularensis* in macrophages, we measured *F. tularensis* replication in J774 cells in the presence and absence of Bref A. Cells were infected with Schu S4-LUX and growth was monitored by measuring luminescence every 30 minutes. We found that *F. tularensis* replication was significantly reduced in Bref A-treated J774 cells (Figure 5C, 5D), and growth was significantly rescued in Bref A treated cells by the addition of amino acids (Figure 5C, 5D). Bref A cytotoxicity was comparable regardless of amino acid supplementation, indicating that the increase in bacterial replication was not due to decreased eukaryotic cell cytotoxicity in amino acid treated cells (Figure S3C). The ability of amino acids to rescue bacterial replication in Bref A-treated cultures indicates that Bref A affects *F. tularensis* nutrient availability. This result is consistent with the conclusion that ATG5-independent autophagy provides nutrients that support *F. tularensis* growth in macrophages as well as in MEFs.

**Figure 6:**
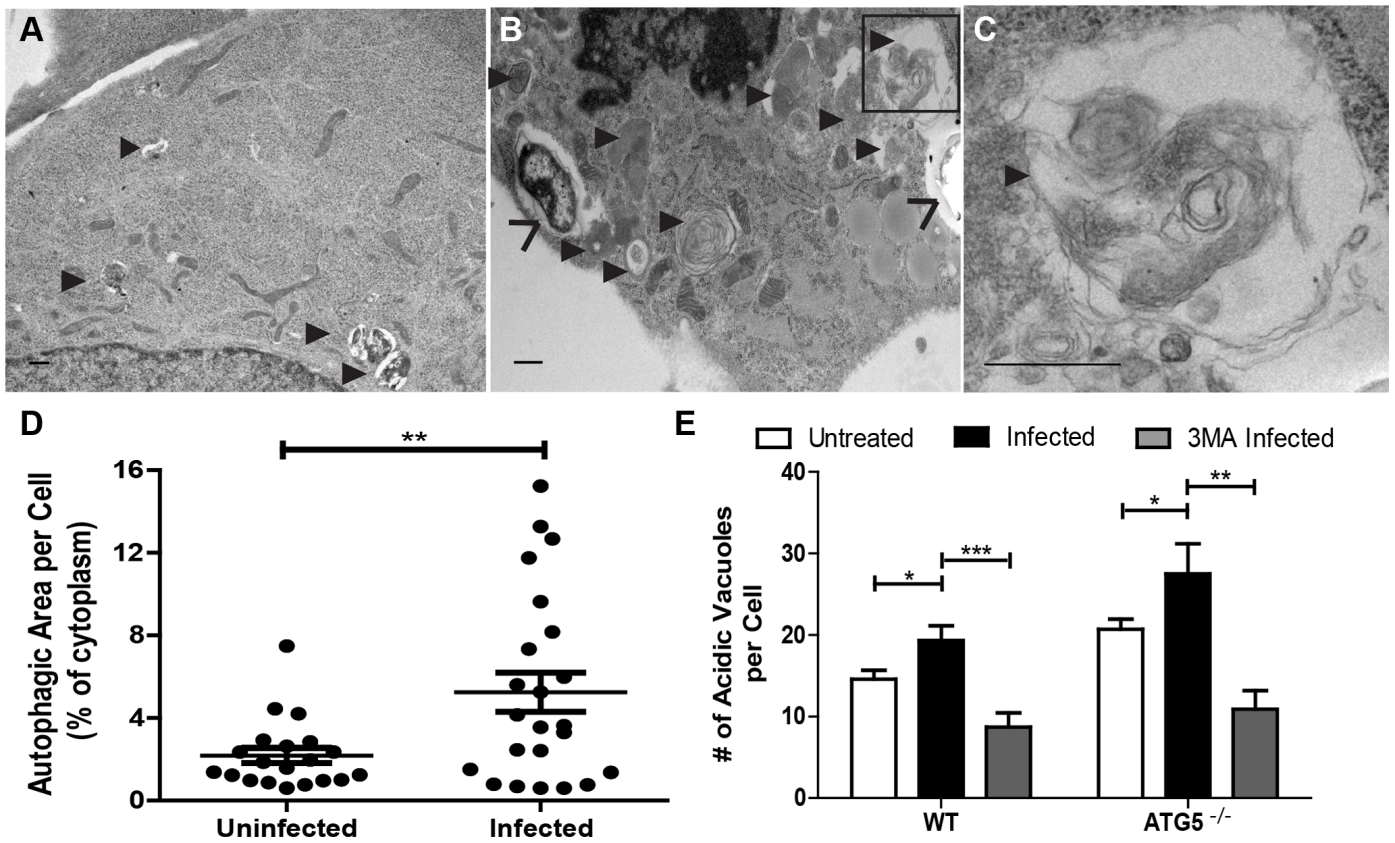
*F. tularensis* induces ATG5-independent autophagy in infected cells. Representative transmission electron micrographs of (**A**) uninfected and (**B**) *F. tularensis* infected ATG5^−/−^ MEFs. (**C**) Higher magnification of representative infected MEF. *F. tularensis* is depicted with open faced arrows (>) and autophagosomes with solid arrows (▸). All scale bars represent 0.5 um. (**D**) The percentage of cytoplasm that is autophagic in ATG5^−/−^ MEFs in uninfected and infected cells (• represents 1 cell, n≥20 per sample). (**E**) The number of acidic vacuoles per cell in wild type and ATG5^−/−^ MEFs. MEFs were uninfected, infected, or infected and treated with 3MA (mean +/− SEM, n>30 cells per sample from 6 independent experiments). (* p<0.05, **p<0.01, ***p<0.001).

We wanted to determine the extent to which autophagosomes are formed during *F. tularensis* infection, and the spatial relationship between the bacteria and autolysosomes in ATG5^−/−^ cells. Analysis of transmission electron microscopy (TEM) micrographs revealed that autophagic vacuoles constituted a greater percentage of the cytoplasm in *F. tularensis* infected as compared to uninfected ATG5^−/−^ MEFs (Figure 6A–D) confirming that autophagy is induced in ATG5^−/−^ MEFs.

Since morphological analysis of autophagic structures by TEM is inexact, we used fluorescence confocal microscopy as a secondary means to identify acidified autophagic vacuoles in infected MEFs. We stained and quantified the number of LysoTracker Red positive acidic vacuoles in infected and uninfected ATG5^−/−^ MEFs. There were significantly more acidic vacuoles in the infected ATG5^−/−^ MEFs as compared to uninfected ATG5^−/−^ MEFs (Figure 6E). LysoTracker Red can also stain other acidic vacuoles including lysosomes and phagosomes. However, the increased number of acidic vacuoles found in infected wild type and ATG5^−/−^ MEFs as compared to uninfected and 3MA treated infected control cells strongly argues that the increase in acidic vacuoles correlate with an increase in autophagic vacuoles. Combined with the morphological analysis of the infected-cell vacuoles by TEM this data demonstrates that *F. tularensis* induced ATG5-independent autophagy in infected cells.

### Neither canonical autophagy nor xenophagy are induced during *F. tularensis* intracellular replication

**Figure 7:**
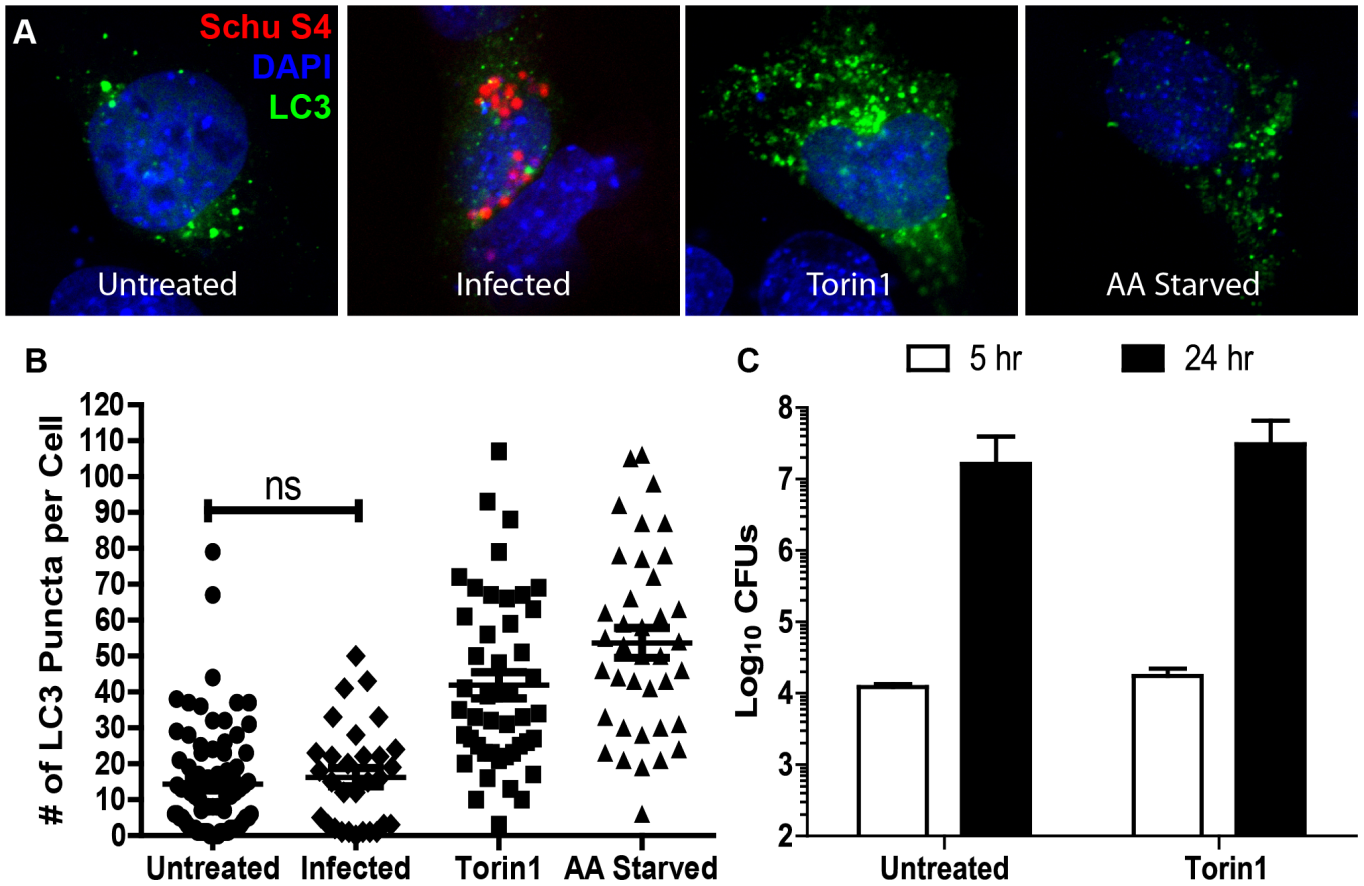
*F. tularensis* does not induce canonical autophagy during late logarithmic phase of intracellular growth. (**A**) Representative confocal microscopy images depicting LC3-GFP transfected MEFs with the indicated treatments. DAPI is represented in blue, LC3 in green, and *F. tularensis* in red. (**B**) The number of GFP puncta in LC3-GFP transfected MEFs that were untreated, infected for 16 hours, Torin1 treated for 2 hours, or amino acid starved for 2 hours (mean +/− SEM, n>30 cells per sample, 4 independent experiments). (**C**) Number of intracellular *F. tularensis* 5 and 24 hours post inoculation of untreated or Torin1 treated MEFs (mean +/− SD, 3 independent experiments). (ns p>0.05).

The slight but statistically significant increase in *F. tularensis* growth observed in ATG5^−/−^ MEFs suggested that canonical autophagy may be induced in infected cells and exert some control over bacterial growth. It is also possible that in addition to destroying the bacteria, canonical autophagy could serve as a redundant mechanism for nutrient acquisition. To determine if canonical autophagy was induced in addition to ATG5-independent autophagy during infection with *F. tularensis*, we analyzed infected MEFs that were transiently transfected with a GFP-LC3 plasmid for an increase in GFP-LC3 puncta. LC3 puncta formation is stimulated by canonical autophagy; however, ATG5-independent autophagy does not induce LC3 puncta formation [24], [26]. LC3 puncta levels were unchanged in infected compared to uninfected MEFs at 16 hours post inoculation, whereas both the amino acid starvation and Torin1 controls conferred an increase in LC3 puncta (Figure 7A, B). Thus, it appears that canonical autophagy remained at basal levels in *F. tularensis* infected cells during late stages of infection.

To determine if induction of canonical autophagy would either increase bacterial clearance or generate additional nutrients that support bacterial replication, we artificially induced autophagy throughout infection with the mTOR inhibitor Torin1. Torin1 treatment throughout infection had no impact on *F. tularensis* intracellular survival or growth in MEFs (Figure 7C). Thus, *F. tularensis* evades destruction by canonical autophagy and increased canonical autophagy did not benefit *F. tularensis* intracellular replication.

*F. tularensis* induces ATG5-independent autophagy while canonical autophagy remains at basal levels during infection. Little is known about the functional differences between canonical and ATG5-independent autophagy. However, xenophagy is known to occur via canonical autophagy whereas xenophagy via ATG5-independent autophagy has not been addressed. In canonical autophagy, cytosolic pathogens including chloramphenicol treated *F. tularensis* are targeted for xenophagy when bound to p62/SQSTM1 and polyubiquitin [17], [27]–[29]. We therefore investigated the role of polyubiquitin and p62/SQSTM1 in ATG5-independent autophagy induction in *F. tularensis* infected cells.

**Figure 8:**
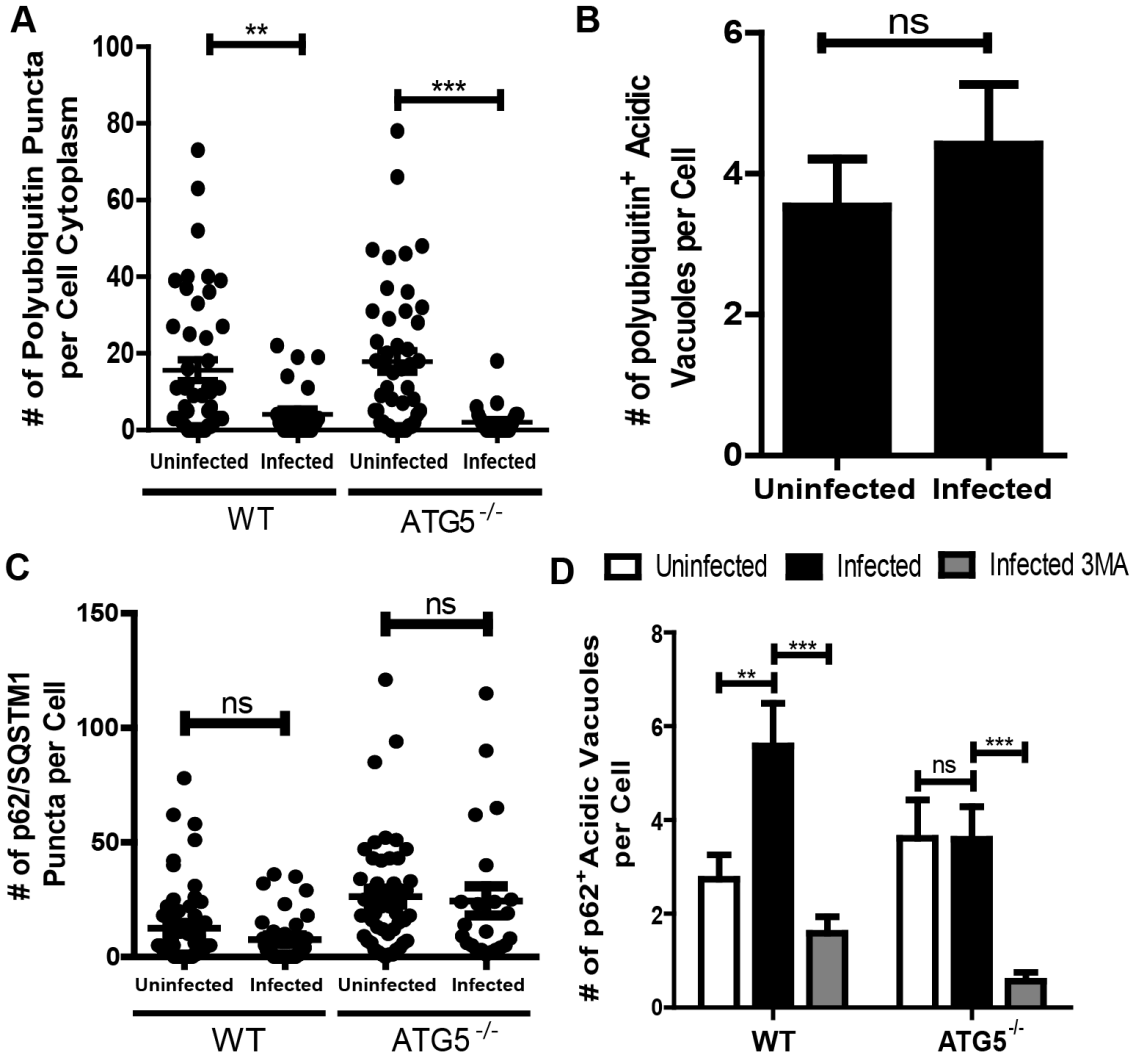
p62/SQSTM1 and polyubiquitin puncta in *F. tularensis* infected cells. (**A**) The number of polyubiquitin puncta in the cytoplasm of uninfected and *F. tularensis* infected wild type and ATG5^−/−^ cells 16 hours post inoculation (• represents 1 cell, n≥25 per sample, 3 independent experiments). (**B**) The number of acidic vacuoles that co-localized with a polyubiquitin puncta per cell in uninfected and *F. tularensis* infected wild type MEFs 16 hours post inoculation (mean +/− SEM, n>25 cells per sample, 3 independent experiments). (**C**) The numbers of p62/SQSTM1 puncta per cell in uninfected and *F. tularensis* infected wild type and ATG5^−/−^ cells 16 hours post inoculation (• represents 1 cell, n≥35 per sample, 3 independent experiments). (**D**) The numbers of p62 positive acidic vacuoles in wild type or ATG5^−/−^ MEFs that were untreated, infected, or infected and treated with 3MA where infected samples were enumerated16 hours post inoculation (mean +/− SEM, n>30 cells per sample, 3 independent experiments). (ns p>0.05, * p<0.05, **p<0.01, ***p<0.001).

There was a significant decrease in the number of polyubiquitin puncta in the cytoplasm of infected wild type and ATG5^−/−^ MEFs as compared to uninfected MEFs (Figure 8A). If polyubiquitin was degraded upon ATG5-independent autophagy induction, we would expect a corresponding increase in co-localization between polyubiquitin and acidic vacuoles in infected cells. However, the number of acidic vacuoles co-localizing with polyubiquitin in uninfected cells (15.2%+/−2.2%) and infected cells (20.0%+/−3.5%) was not significantly different (n>25 cells, mean +/− SEM) (Figure 8B). These data indicate that the decrease in polyubiquitin aggregates in infected cells was independent of autophagy.

In addition, there were similar numbers of p62/SQSTM1 puncta in infected MEFs compared to uninfected MEFs (Figure 8C, S5C–S5E). Interestingly, although there were similar total numbers of p62/SQSTM1 puncta, there was increased co-localization of p62/SQSTM1 with acidic vacuoles in infected wild type MEFs. However, there was no difference in p62/SQSTM1 co-localization between uninfected and infected ATG5^−/−^ MEFs (Figure 8D). The increased co-localization of p62/SQSTM1 with acidic vacuoles may indicate that some basal level of xenophagy is occurring in an ATG5-dependent manner, which is consistent with the increase in bacterial replication that we observed in ATG5^−/−^ MEFs. Taken together, these data indicate that *F. tularensis* induced ATG5-independent autophagy is not associated with polyubiquitin, LC3B, or p62/SQSTM1.

### *F. tularensis* is adjacent to autophagic vacuoles

A recent study demonstrated that *Salmonella enterica* associates with ubiquitinated aggregates that are degraded by autophagy [30]. Although these aggregates likely target *S. enterica* for degradation rather than supplying nutrients, these data suggest that mechanisms exist which target autophagosomes to bacteria or vice versa. We hypothesized that *F. tularensis* may recruit autophagic vacuoles, resulting in bacteria localizing in close proximity to autophagosomes to facilitate bacterial nutrient acquisition. Indeed, *F. tularensis* was frequently found within 250 nm of autophagic vacuoles in both ATG5^−/−^ MEFs and J774 cells as determined by TEM (Figure S6A, S6B). Indeed, 25.8+/−4.0% (average +/− SEM) of the autophagic vacuoles in ATG5^−/−^ MEFs were also within 250 nm of a bacterium.

We confirmed the TEM results using confocal microscopy. Since ATG5-independent autophagy does not appear to require ubiquitination or any known target marker, we were limited to examining the relationship between bacteria and acidified vacuoles. Infected cells were stained with LysoTracker Red and Z-stacks from infected cells were analyzed by confocal microscopy. 28.0%+/−3.7% of bacteria in wild type MEFs and 35.1%+/−5.1% of bacteria in ATG5^−/−^ MEFs were within 250 nm of an acidic vacuole (Average +/− SEM, n>10 cells) (Figure S6 C–H). At least 1 bacterium was within 250 nm of an acidic vacuole in every cell. The number of bacteria within 250 nm of an acidic vacuole was significantly lower in 3MA treated MEFs compared to the untreated MEFs (p = .01) (Figure S3 H). These data suggest that *F. tularensis* may recruit or traffic to autophagic vacuoles. Further investigation may reveal that not only autophagy induction, but also proximity to an autophagic vacuole contributes to *F. tularensis* nutrient acquisition.

## Discussion

Intracellular pathogens have evolved to thrive within the hostile nutrient-limited host cell environment. Successful pathogens disarm or avoid innate and adaptive immune responses while simultaneously extracting carbon and energy sources to support their proliferation. Autophagy is a highly conserved degradation process that serves a multitude of functions including cell development, stress response and resistance to cytoplasmic pathogens. Herein we investigated the interaction between *F. tularensis* and the host cell autophagy response. Our results demonstrate that ATG5-independent autophagy is triggered in *F. tularensis* infected cells and that intracellular bacterial replication was enhanced by this process. Furthermore, *F. tularensis* can replicate in cells when there are no amino acids present in the media, indicating that *F. tularensis* obtains all of the amino acids necessary to fulfill its 13 amino acid auxotrophies from the host cell through processes such as autophagy. *F. tularensis* acquires amino acids, and possibly other nutrients, via autophagy. These nutrients are then used for both energy and protein synthesis, although decreased bacterial replication in ATG5-independent autophagy deficient cells is primarily due to a lack of available energy. Autophagy derived nutrients are necessary for optimal *F. tularensis* replication, but *F. tularensis* still replicated in cells with decreased ATG5-independent autophagy. This indicates that *F. tularensis* uses other nutrient acquisition strategies in conjunction with ATG5-independent autophagy to supply nutrients for rapid and efficient proliferation.

Rapid bacterial proliferation requires readily available and abundant carbon and energy sources, commodities that are typically limited in the eukaryotic cell environment. Intracellular pathogens must acquire all required nutrients from the host cell, but the strategies that these pathogens employ to accomplish this task are only beginning to be characterized and vary widely between pathogens [9], [10], [31]–[33]. For example, *Legionella pneumophila* uses the byproducts of host proteosomal degradation rather than autophagy to obtain amino acids for energy [31]. Dengue virus growth is supported by autophagy mediated release of lipids while autophagosome formation increases nutrient availability for *Anaplasma phagocytophilum* [9], [10]. It is likely that other intracellular pathogens that successfully avoid autophagic destruction benefit from the nutrients that are released by this process. Thus, autophagy subversion through various means may be a more common strategy for pathogens to acquire nutrients from the host than previously thought.

The conclusion that autophagy derived amino acids were sufficient to rescue intracellular growth was supported by the fact that the absence of amino acids in tissue culture media did not appreciably affect *F. tularensis* intracellular replication. Thus, host cell amino acid import was not required to support bacterial growth. This result would seem to contradict the recent observation that knocking down expression of the amino acid transporter SLC1A5 decreases *F. tularensis* LVS growth approximately 2-fold [32]. LVS is an attenuated *F. tularensis* vaccine strain that, like fully virulent *F. tularensis*, grows within macrophages and other cell types, but is significantly less virulent than *F. tularensis* and other wild type *F. tularensis* strains in humans and animal models of infection. Unlike *F. tularensis* Schu S4, we found that LVS intracellular growth was significantly impaired in ATG5^−/−^ MEFs and growth in these cells was restored by supplying excess amino acids, implying that LVS harvests nutrients via ATG5-dependent autophagy or another ATG5-dependent mechanism (data not shown). It is therefore likely that LVS is less reliant on ATG5-independent autophagy to support efficient intracellular growth. It is also possible that SLC1A5 contributes to the export of free amino acids out of autolysosomes thereby making autophagy derived amino acids available to the cytoplasmic bacteria. Amino acid transporters export amino acids from autolysosomes to the cytosol in *Saccharomyces cerevisiae*, and a similar system likely exists in mammalian cells [34]. This latter possibility highlights the fact that currently little is known about how free amino acids derived from autophagic degradation of host proteins are transported within eukaryotic cells.

Canonical autophagy destroys several different pathogens, including replication deficient and chloramphenicol treated *F. tularensis* [17]. The slight increase in bacterial replication in ATG5 −/− MEFs compared to wild type MEFs supports the notion that canonical autophagy can degrade wild type bacteria in MEFs, although this may be cell type specific as there is no difference in *F. tularensis* replication between wild type and ATG5^−/−^ bone marrow derived macrophages [17]. Also, induction of autophagy by starvation or Torin1 treatment did not reduce bacterial replication. Surprisingly, although we observed mTOR inhibition in J774 cells and autophagy induction in ATG5^−/−^ MEFs, our results suggest that canonical autophagy is either at or close to basal levels 16 hours post inoculation. Our results suggest that *F. tularensis* suppresses canonical autophagy downstream of mTOR or that mTOR is inhibited in ATG5-independent autophagy and other signals help determine which autophagy pathway is induced.

In contrast to xenophagy via canonical autophagy, ATG5-independent autophagy is involved in the lifecycle of two other intracellular bacterial pathogens. *Mycobacterium marinum* and *Brucella abortus* are each sequestered into an autophagosome-like structure via an ATG5-independent pathway as part of their intracellular lifecycles [8], [35]. It is unclear why *M. marinum* is sequestered, but bacterial sequestration by autophagy appears to be part of the *B. abortus* intracellular lifecycle and may benefit the bacteria by increasing cell to cell spread rather than providing nutrients [8], [35]. Both of these interactions with ATG5-independent autophagy are different from that of *F. tularensis*. What remains to be determined is if this difference is due to bacterial manipulation, if there are multiple ATG5-independent autophagy pathways, or if there are different functions for the same ATG5-independent autophagy pathway. Unfortunately, there is little information about how the various autophagy pathways are functionally different. We found that ATG5-indepdendent autophagy, unlike canonical autophagy, does not appear to use two proteins associated with xenophagy during infection. Further characterization of how xenophagy and ATG5-independent autophagy are associated may reveal why certain pathogens induce ATG5-independent autophagy.

Little is known about how ATG5-independent autophagy is induced or the role that it plays in a healthy eukaryotic cell, let alone during pathogenesis. However, there appears to be distinct benefits for certain pathogens to induce ATG5-independent autophagy over the canonical autophagy pathway. Determining how this pathway is induced in *F. tularensis* infected cells may give us insight as to how different autophagy pathways are initiated and how these pathways differentially impact intracellular pathogen survival and growth.

## Materials and Methods

### Bacteria and plasmids

*Francisella tularensis* subsp. *tularensis* Schu S4 was obtained from Biodefense and Emerging Infections Research Resources Repository. For inoculation of eukaryotic cells Schu S4, Schu DSred, Schu S4-GFP [15] and Schu S4 – LUX (plasmid from [22]) were each grown initially on chocolate agar supplemented with 1% isovitalex then overnight in Chamberlain's defined broth media (CDM).

### Cell culture

J774A.1 macrophage-like cells (J774) cells were maintained in 4.5 g/L glucose Dulbecco's minimal essential media (DMEM) with 10% FBS and supplemented with L-glutamine and sodium pyruvate. Mouse embryonic fibroblasts (MEFs) were maintained in 4.5 g/L glucose DMEM with 10% FBS. For treatment of MEFs without amino acids, DMEM with 4.5 g/L glucose was made following the ATCC DMEM protocol without adding amino acids and supplemented with 10% dialyzed FBS.

Human monocyte derived macrophages (hMDMs) were obtained by isolating peripheral blood mononuclear cells (PBMCs) from blood via ficoll gradient centrifugation. PBMCs were cultured for 2 hours in RPMI with 10% FBS and then washed to remove non-adherent cells. The adherent cells were cultured for 2 weeks in RPMI containing 10% FBS and 3 ng/ml GM-CSF (Biolegend). The media was replaced every 2 days. Experiments were performed using PBMCs isolated from peripheral blood from 2 healthy volunteers who gave informed, written consent following a protocol approved by the Institutional Review Board for human volunteers at University of North Carolina at Chapel Hill. Peripheral blood was obtained specifically for these experiments.

Stable Beclin-1 knockdown (TRCN0000087289 or TRCN0000087291) and scramble cell lines were generated by transducing MEFs with lentivirus encoding each shRNA. Cells were propagated in media containing 1 µg/ml puromycin for 2 weeks prior to the first experiment to select for transduced cells. Concurrent with the first experiment and last intracellular bacterial proliferation assay in the knockdown cell lines, mRNA was harvested from the transduced cells, subjected to reverse transcription, and was analyzed by quantitative RT-PCR to determine the amount of Beclin-1 mRNA present in each sample. The results were normalized to a GAPDH control. Primer sequences in are in Table S1.

### Drug treatments

3-methyladenine (10 mM) (Sigma), bafilomycin A(1) (200 nM) (Sigma), and chloroquine (160 µM) (Sigma) were each added with 25 µg/ml of gentamicin to the MEFs 3 hours post bacterial inoculation. Brefeldin A (17 µM) (Sigma) was added to J774 cells 3 hours post inoculation. Torin1 (250 nM) (Tocris Biosciences) was added overnight prior to inoculation and maintained throughout the infection. The excess amino acid mixture (12 mM L-amino acids containing aspartic acid, arginine, cysteine, histidine, isoleucine, leucine, lysine, methionine, proline, serine, threonine, tyrosine, and valine), L- serine (15 mM) or pyruvate (18 mM) were added at the same time as the inhibitors. All media was brought to a pH of 7.5.

Inhibitor cytotoxicity in MEFs was determined using a Live/Dead Fixable Green Dead Cell Stain kit (Invitrogen) following the manufacturer's instructions. Drugs were placed on cells for the same duration they would be on cells during infection (21 hours for Baf and CQ, 29 hours for 3MA). Percent cytotoxicity by flow cytometry was determined by gating. Cytotoxicity of *F. tularensis* in J774 cells 16 hours post inoculation was determined by testing the amount of lactate dehydrogenase (LDH) in the supernatant with a CytoTox-Glo cytotoxicity kit (Promega) following the manufacturer's instructions. Percent cytotoxicity was determined based on media and digitonin treated controls. Brefeldin A cytotoxicity was determined 21 hours post treatment using an *In vitro* Toxicology Assay Kit (Sigma) to measure LDH release from J774 cells.

### Intracellular growth assays

MEFs were plated at 2×10^5^ cells per well in 24 well tissue culture treated plates and grown overnight. MEFs were inoculated with a multiplicity of infection (MOI) of 100 with wild type Schu S4. The media was removed 3 hours post inoculation and replaced with media containing 25 µg/ml of gentamicin to inhibit the growth of any remaining extracellular bacteria. MEFs were lysed by vortexing for 1 minute and the lysates were serially diluted and plated on chocolate agar to calculate the number of intracellular bacterial cells at the indicated times.

hMDM cells were inoculated with an MOI of 100 wild type Schu S4 in RPMI containing 10% FBS. At 2 hours post inoculation, the media was replaced with media containing 10 µg/ml of gentamicin. At 4 hours post inoculation, the media was replaced with media that did not contain gentamicin. Intracellular bacteria were quantified as described previously.

Bacterial intracellular growth kinetics was calculated by measuring luminescence of Schu S4 – LUX infected MEFs or J774 cells. MEFs and J774 cells were plated at 5×10^4^ cells per well in 96 well black wall clear bottom polystyrene plates (Corning) the night before infection. Each well was inoculated at an MOI of 100 with Schu S4- LUX and treated with gentamicin and inhibitors as described above. Luminescence was measured every 30 minutes using an Infinite M200 Pro plate reader (Tecan) maintaining constant 37°C temperature and 5% carbon dioxide.

All intracellular growth assays were performed in triplicate for each independent experiment. All of the inhibitors were added 3 hours post inoculation to reduce the impact of the inhibitors on *F. tularensis* phagosomal escape.

### Growth curves

Bacterial growth curves of broth cultures were generated by measuring the optical density at 600 nm (OD600 every 15 minutes) using an Infinite M200 Pro plate reader (Tecan) maintaining constant temperature (37°C). To test toxicity of each drug on Schu S4, the bacteria were grown in CDM overnight, and then diluted to an OD600 of 0.05 in CDM containing the indicated inhibitors. CDM glucose substitution media were made without added glucose and 30 mM of the defined amino acid or carbon source. 50 mM MES buffer was added to all CDM media in the glucose substitution experiments.

### Fluorescence microscopy

For confocal fluorescent microscopy images depicting the number of bacteria in drug treated cells, MEFs were plated at 1×10^4^ cells per well in an 8 well chamber slide (Nunc) and grown overnight. MEFs were inoculated at a MOI of 100 with Schu S4-GFP or Schu S4- DSred and treated with 25 µg/ml of gentamicin as described above. At the indicated time post inoculation, the MEFs were washed and fixed with 4% paraformaldehyde for 15 minutes and then washed again in PBS. To stain the plasma membrane, 10 µg/ml of AF647 conjugated wheat germ agglutinin (Invitrogen) was added to the fixed cells for 5 minutes and then washed away. DAPI containing mounting media (Vector Shield) was added to the slides to identify the nucleus.

Infection frequency was determined by fixing GFP infected MEFs 5 or 6 hours post inoculation and comparing the number of cells containing green puncta to the total number of cells completely within the field of view.

To quantify LC3B puncta, GFP-LC3 MEFs were generated by transfecting MEFs attached to an 8 well chamber slide (Nunc) with an eGFP-LC3 plasmid (Addgene plasmid 21073) [26]. 18 hours after transfection, the media was replaced with fresh media for one hour. After one hour, the cells were either infected with Schu-DSred or placed in fresh media. 3 hours post inoculation, the media in all wells was replaced with media containing 25 µg/ml gentamicin. 14 hours post inoculation, Torin1 or media lacking amino acids was added to the appropriate wells. The cells were fixed as above and stained with a mouse anti-GFP antibody (1∶250 dilution, Millipore) followed by an AF488 anti-mouse secondary antibody (Invitrogen) as previously described.

To quantify acidic vacuoles and determine co-localization with polyubiquitin and p62, MEFs were initially prepared as described above but were incubated for 2 hours in the presence of 150 ng/ml of LysoTracker red (Invitrogen) beginning at 14 hours post inoculation. The cells were washed and MEF media was added for an additional 10 minutes at 16 hours post inoculation. The cells were fixed in 4% paraformaldehyde and treated with 10 mM ammonium chloride following fixation. The MEFs were incubated with a polyubiquitin antibody (1∶1000 dilution, Enzo Life Sciences) or a p62/SQSTM1 primary antibody (1∶250 dilution, Abnova) followed by an AF647 conjugated anti-mouse secondary antibody (Invitrogen). DAPI containing mounting media (Vector Shield) was added to the slides to identify the nucleus. Images were acquired using a Zeiss 700 confocal laser scanning microscope (Carl Zeiss SMT, Inc.). Image acquisition, contrast adjustments, and cropping were all performed using Zen 2011 (Carl Zeiss SMT, Inc.).

Acidic vacuoles, p62, and polyubiquitin puncta were quantified by setting thresholds using ImageJ [36]. Only polyubiquitin puncta outside of the nucleus were counted. Co-localization of p62 or ubiquitin puncta with acidic vacuoles was determined by manual counting overlap. Any acidic vacuole or bacteria that overlapped any portion of the puncta was considered to co-localize.

To determine the distance between acidic vacuoles and *F. tularensis*, Z-stacks of LysoTracker red stained cells were taken using a Flow View 500 confocal laser scanning microscope (Olympus America). The distance between the bacteria and the acidic vacuoles was determined using ImageJ [36]and Corsen [37], following the protocols described in Jourdren et al. Additional protocol information and ImageJ plug-ins were available at http://transcriptome.ens.fr/corsen. The distance between objects was measured from the surface of the bacteria to the closest surface of the nearest acidic vacuole. To decrease the impact of noise, acidic vacuoles and bacteria with a volume of less than 0.05 µm (as determined by the Corsen program) were not included in the analysis.

### Radiolabel experiments

To monitor transfer of amino acids from the host cell to *F. tularensis*, 4×10^5^ MEFs were incubated in cysteine and methionine free DMEM containing 10% dialyzed FBS and 0.125 mC of S^35^ radiolabelled cysteine and methionine (EasyTag Express ^35^S, Perkin-Elmer) for 18 hours. 10 µg/ml of cycloheximide was added with the radiolabel in the indicated sample. The MEFs were then washed once and then incubated with DMEM containing 10% FBS for 2 hours. DMEM contains in excess of 100,000 times more cysteine and methionine than the initial radiolabel. The MEFs were then inoculated with *F. tularensis* Schu S4 at an MOI of 100 for 3 hours in fresh media. At 3 hours post inoculation, the media was replaced with media containing 25 µg/ml of gentamicin and either Baf or 3MA, as indicated, and supplemented with either a 12 mM amino acid mixture or 18 mM serine. The cells were washed in PBS, scraped from the plate, and lysed by vortexing the in PBS 16 hours post inoculation. The cell lysates were mixed with streptavidin coated magnetic beads (Solulink) that were pre-bound to biotinylated anti-*F. tularensis* lipopolysaccharide antibody (US biological). The anti-*F. tularensis* LPS antibody was biotinylated using a Biotin-xx protein labeling kit following the manufacturer's instructions (Invitrogen). The bead lysate mixture was incubated at room temperature for 20 minutes and then washed three times on a magnet. After the final wash, an equal volume of beads was added to 20% trichloroacetic acid (TCA) to make a final concentration of 10% TCA. The TCA mixture was mixed with an equal volume of 5% BSA and spun to pellet the TCA insoluble fraction. The TCA soluble fraction was removed and the TCA insoluble fraction was resuspended in PBS, added to scintillation fluid, and the number of counts was measured. An aliquot of the sample after the final wash was plated on chocolate agar to determine the number of bacteria present. The percent of radiolabel that was incorporated into *F. tularensis* was calculated by dividing the radiolabel counts from samples taken immediately prior to infection by the difference between the infected and uninfected samples.

To evaluate host protein degradation, J774 cells were radiolabeled for 24 hours, chased with non-radioactive media, inoculated and treated with gentamicin as described above. At 16 hours post inoculation, the cells were washed in PBS and lysed in RIPA buffer. The lysate was spun immediately to pellet the insoluble fraction. The soluble fraction was harvested and added to an equal volume of 20% TCA. The TCA insoluble fraction was then prepared and quantified as above.

### Electron microscopy

Uninfected and Schu S4 infected J774 cells or ATG5^−/−^ MEFs were maintained on small plastic tissue culture dishes. 25 µg/ml of gentamicin was added 2 hours post inoculation for J774 cells and 3 hours post inoculation for MEFs. 16 hours post inoculation the cells were fixed for 1 hour at room temperature in 2% paraformaldehyde, 0.5% glutaraldehyde in 0.15 M sodium phosphate buffer at pH 7.4. The cells were then rinsed in buffer and post-fixed with 0.5% osmium tetroxide/0.15 M sodium phosphate buffer, pH 7.4, for 10 minutes.

TEM samples for J774 cells were prepared similarly, although the cells were post-fixed for 1 hour in 1% osmium tetroxidein 0.15 M sodium phosphate buffer at pH 7.4 and then stained *en bloc* with 2% aqueous uranyl acetate for 20 minutes.

Both fixed samples were dehydrated in ethanol (30%, 50%, 75%, 100%, 5 minutes each step) and infiltrated and embedded in L.R. White Resin (Electron Microscopy Sciences). The dehydrated samples were sectioned en face (parallel to the substrate) at 70 nm, mounted on 200 mesh nickel grids, and post-stained with 4% uranyl acetate followed by Reynolds' lead citrate. Samples were observed with a LEO EM910 transmission electron microscope operating at 80 kV (Carl Zeiss SMT, Inc.) and digital images were acquired using a Gatan Orius SC1000 CCD Digital Camera with Digital Micrograph 3.11.0 (Gatan).

### Western blot analysis

For the phospho- S6 ribosomal protein western blots, J774 cells were inoculated with Schu S4 at an MOI of 100 and treated with 25 µg/ml gentamicin 2 hours post inoculation. The uninfected sample had media replaced and media containing gentamicin added at the same times as infected samples. The uninfected samples were harvested 24 hours post inoculation. At the indicated times, cells were lysed by adding water containing phosphatase (Roche) and protease inhibitor cocktails (Pierce) and vortexing. The lysates were filtered through two 0.22 µm filters, separated on an SDS-PAGE gel under reducing conditions and then transferred to a nitrocellulose membrane. The membranes were probed with rabbit anti- S6 ribosomal protein or rabbit anti- phospho S6 ribosomal protein (Ser 235/236). All primary antibodies were obtained from Cell Signaling Technologies. Membranes were then probed with a horse radish peroxidase conjugated goat anti-Rabbit IgG (KPL) and bands were detected using an ECL Western Blotting Detection Kit (GE Life Sciences). Densitometry analysis was performed using ImageJ and comparing the amount of phosphor S6 ribosomal protein to the total amount of S6 ribosomal protein at the same time point [36]. The densities were then normalized to the uninfected sample.

### Data analysis

Fold change was determined by subtracting each sample from the average of 3 samples taken at 5 hours post inoculation and a Mann-Whitney test was used to determine significance. The rest of the bacterial proliferation assays were pooled across experiments, log_10_ transformed, and then analyzed by a two-tailed Student's t-test were used to measure statistical significance. Significance for bacterial kinetic experiments was performed by pooling the maximum luminescence of each replicate for each experiment and performing a Mann-Whitney test. All luminescence and bacterial proliferation experiments were performed in triplicate in each experiment unless otherwise stated. Statistical significance for the distance measurement between *F. tularensis* and acidic vacuoles was performed using a two tailed Student's t-test on the pooled distance measurements across all 3 experiments for each sample. Significance for radiolabel incorporation into *F. tularensis* was determined by a Mann-Whitney test.

Morphology analysis was performed on the transmission electron micrographs by outlining the whole cell, nucleus, and each bacteria or autophagic vacuole in ImageJ to determine the area of each [36]. Morphology was determined with the aid of the following references [38]–[40]. Any rips in the slice were excluded from this analysis. Each micrograph depicted the nucleus and all infected cells had at least one bacteria present in the slice. The area of cytoplasm was determined by subtracting the area of the nucleus and bacteria from the area of the whole cell. At least 20 cells of each sample were examined and significance was determined by a two tailed Student's t-test.

## Supporting Information

Figure S1 ***F. tularensis*** **replicates to high densities in the host cell cytoplasm.** Representative transmission electron micrographs depicting (**A**) uninfected or (**B**) infected MEFs at 16 hours post inoculation. The scale bars represent 5 µm.
(TIF)

Figure S2 **Autophagy derived nutrients enhance** ***F. tularensis*** **intracellular growth.** Representative intracellular bacterial growth kinetics of *F. tularensis* Schu S4 LUX intracellular growth in untreated and (**A**) 3MA, (**C**) CQ, or (**E**) Baf treated MEFs with or without amino acid supplementation as measured by luminescence (each point represents an average of triplicate wells). Maximum luminescence values from kinetic growth assays for Schu S4 LUX infected J774 cells treated with (**B**) 3MA (10 independent experiments), (**D**) CQ (13 independent experiments), or (**F**) Baf (4 independent experiments). Error bars represent the mean +/− SEM.
(TIF)

Figure S3 **Autophagy inhibitor cytotoxicity.** (**A**) Cytotoxicity of the indicated drugs on MEFs with and without amino acid supplementation (AA) (3 independent experiments, mean +/− SD). (**B**) Representative *F. tularensis* growth curve in Chamberlin's defined media (CDM) containing the indicated drug (curve represents the average of triplicates in a single experiment, 3 independent experiments). (**C**) Cytotoxicity of Brefeldin A on J774 cells with and without amino acid supplementation (AA) (4 independent experiments, mean +/− SD). (**D**) Cytotoxicity of *F. tularensis* on J774 cells at 16 hours post inoculation (3 independent experiments, mean +/− SD).
(TIF)

Figure S4 **Beclin-1 shRNA depletes Beclin-1 mRNA in MEFs.** qRT-PCR quantification of Beclin-1 mRNA in MEFs transduced with a lentivirus encoding a Beclin-1 or scramble shRNA. KD-1 and KD-2 are independently derived lines transduced with different Beclin-1 shRNA's. Results were normalized to GAPDH and are expressed as percent of the scramble control.
(TIF)

Figure S5 ***F. tularensis*** **infection decreases polyubiquitin puncta but increases the number of p62^+^ acidic vacuoles.** Representative fluorescence confocal microscopy images of (**A**) uninfected and (**B**) infected wild type MEFs depicting polyubiquitin. Representative fluorescence confocal microscopy images of (**C**) uninfected, (**D**) infected, or (**E**) infected 3MA treated wild type MEFs stained for p62/SQSTM1. Scale bars represent 10 µm at the low magnification and 2 µm for the higher magnification inset. Nuclei (DAPI) is depicted in blue, GFP-Schu is depicted in green, acidic vacuoles are depicted in red, and polyubiquitin or p62/SQSTM1 are depicted in white.
(TIF)

Figure S6 ***F. tularensis*** **localizes adjacent to autolysosomes.** Representative transmission electron (TEM) microscopy images of Schu S4 (open faced arrows [>]) adjacent to an autophagosome (solid arrows [▸]) in (**A**) J774 cells or (**B**) ATG5^−/−^ MEFs 16 hours post inoculation. The scale bar for the TEM micrograph represents 200 nm. Representative compiled Z-stack images showing the distance (yellow line) between Schu S4 (green) and acidic vacuoles (red) in (**C**) wild type untreated, (**E**) ATG5^−/−^ untreated or (**G**) wild type 3MA treated MEFs 16 hours post inoculation. Scale bars for the 3D images represent 10 µm. The distance between Schu S4 and the closest acidic vacuole in (**D**) untreated wild type (n = 342 bacteria), (**F**) ATG5^−/−^ (n = 401 bacteria) or (**H**) 3MA treated wild type (n = 194 bacteria) MEFs. The distribution histograms are pooled from 3 independent experiments.
(TIF)

Table S1 **Quantitative RT-PCR primer sequences.** Primer sequences for assaying the amount of Beclin-1 or GAPDH mRNA in lentiviral transduced MEFs by qRT-PCR.
(DOCX)
