# Supplementary figures and images for: Francisella tularensis Harvests Nutrients Derived via ATG5-Independent Autophagy to Support Intracellular Growth
Source: PLoS Pathog. 2013 Aug 15;9(8):e1003562. doi: 10.1371/journal.ppat.1003562 (PMC3744417; doi:10.1371/journal.ppat.1003562)

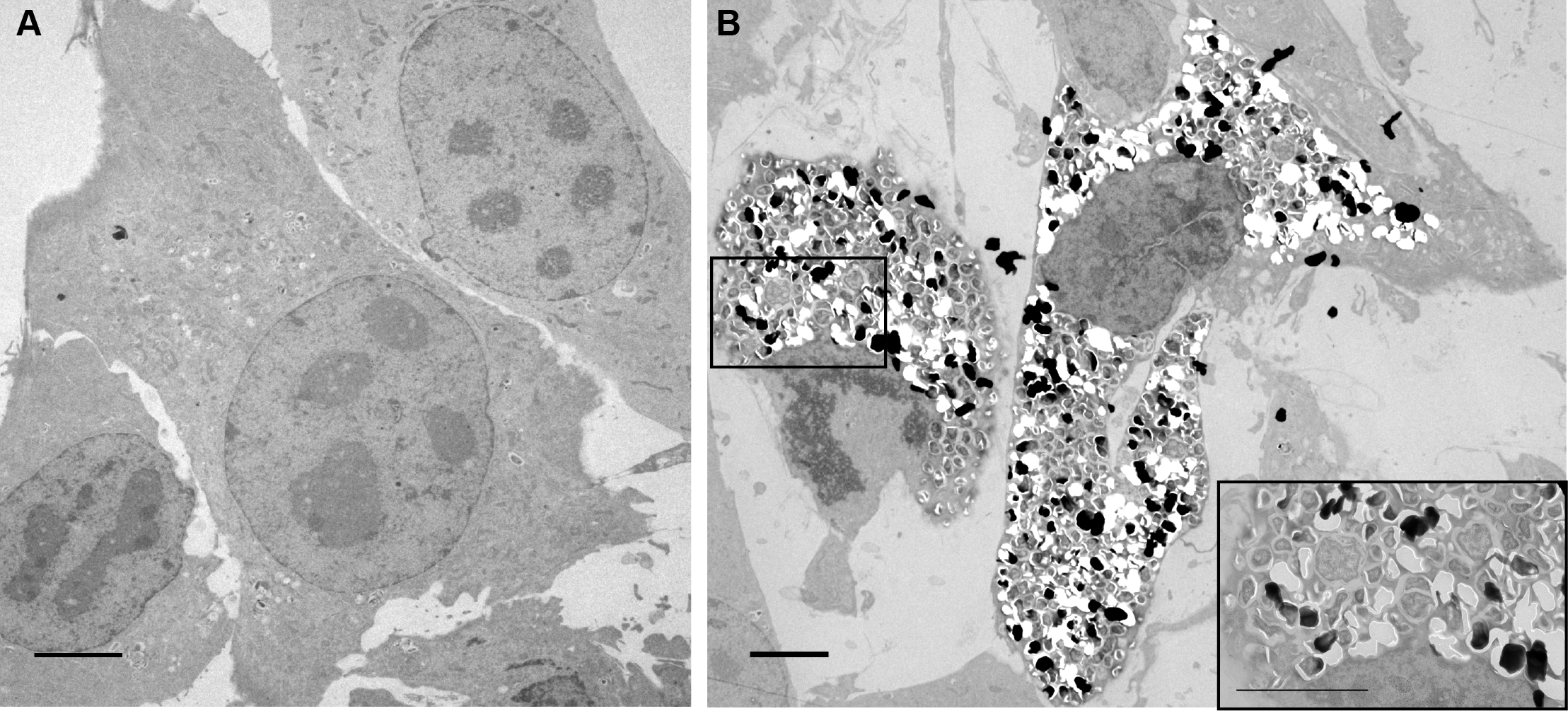

Supplement: Figure S1 — F. tularensis replicates to high densities in the host cell cytoplasm. Representative transmission electron micrographs depicting (A) uninfected or (B) infected MEFs at 16 hours post inoculation. The scale bars represent 5 µm. (TIF) [file ppat.1003562.s001.tif]

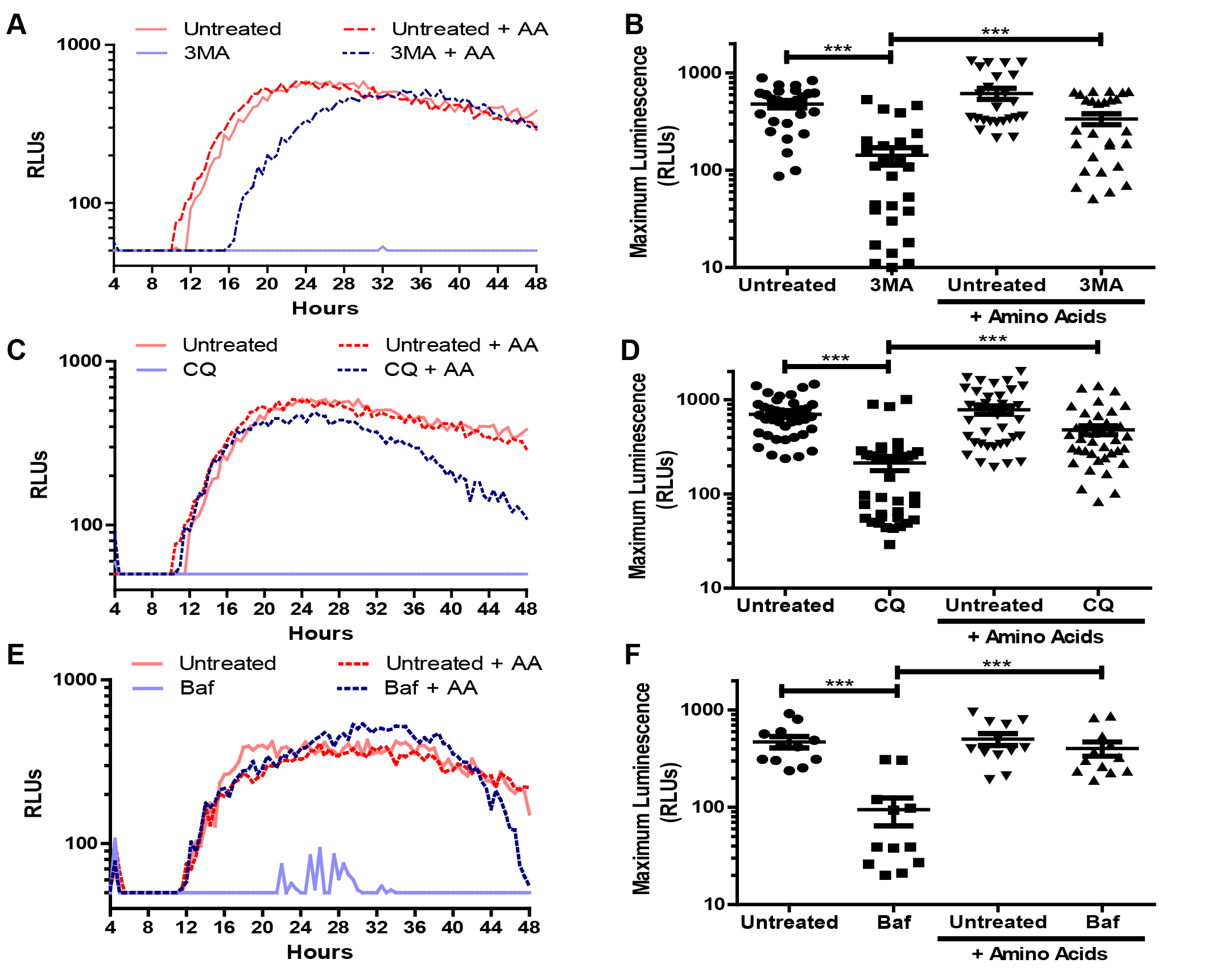

Supplement: Figure S2 — Autophagy derived nutrients enhance F. tularensis intracellular growth. Representative intracellular bacterial growth kinetics of F. tularensis Schu S4 LUX intracellular growth in untreated and (A) 3MA, (C) CQ, or (E) Baf treated MEFs with or without amino acid supplementation as measured by luminescence (each point represents an average of triplicate wells). Maximum luminescence values from kinetic growth assays for Schu S4 LUX infected J774 cells treated with (B) 3MA (10 independent experiments), (D) CQ (13 independent experiments), or (F) Baf (4 independent experiments). Error bars represent the mean +/− SEM. (TIF) [file ppat.1003562.s002.tif]

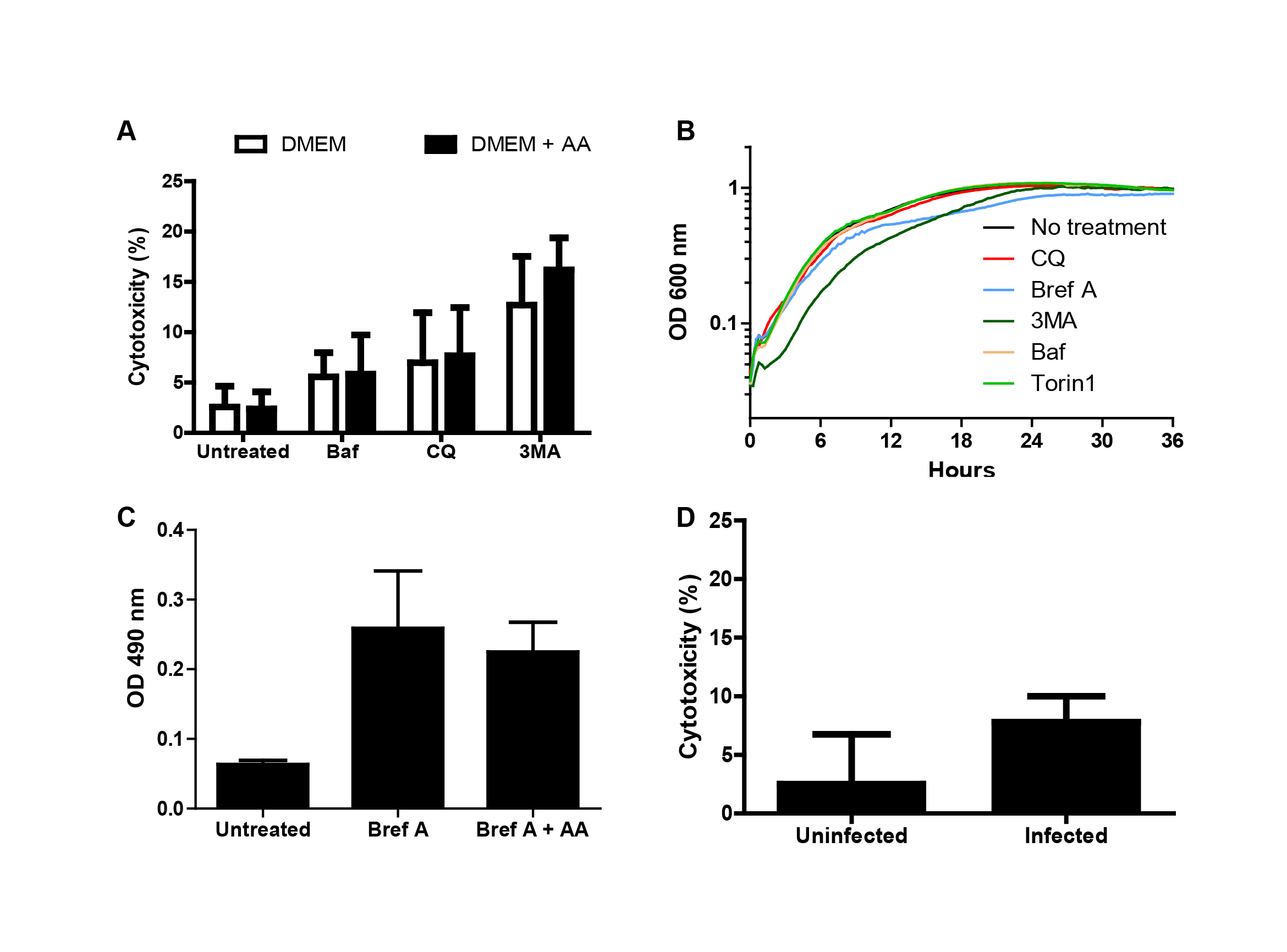

Supplement: Figure S3 — Autophagy inhibitor cytotoxicity. (A) Cytotoxicity of the indicated drugs on MEFs with and without amino acid supplementation (AA) (3 independent experiments, mean +/− SD). (B) Representative F. tularensis growth curve in Chamberlin's defined media (CDM) containing the indicated drug (curve represents the average of triplicates in a single experiment, 3 independent experiments). (C) Cytotoxicity of Brefeldin A on J774 cells with and without amino acid supplementation (AA) (4 independent experiments, mean +/− SD). (D) Cytotoxicity of F. tularensis on J774 cells at 16 hours post inoculation (3 independent experiments, mean +/− SD). (TIF) [file ppat.1003562.s003.tif]

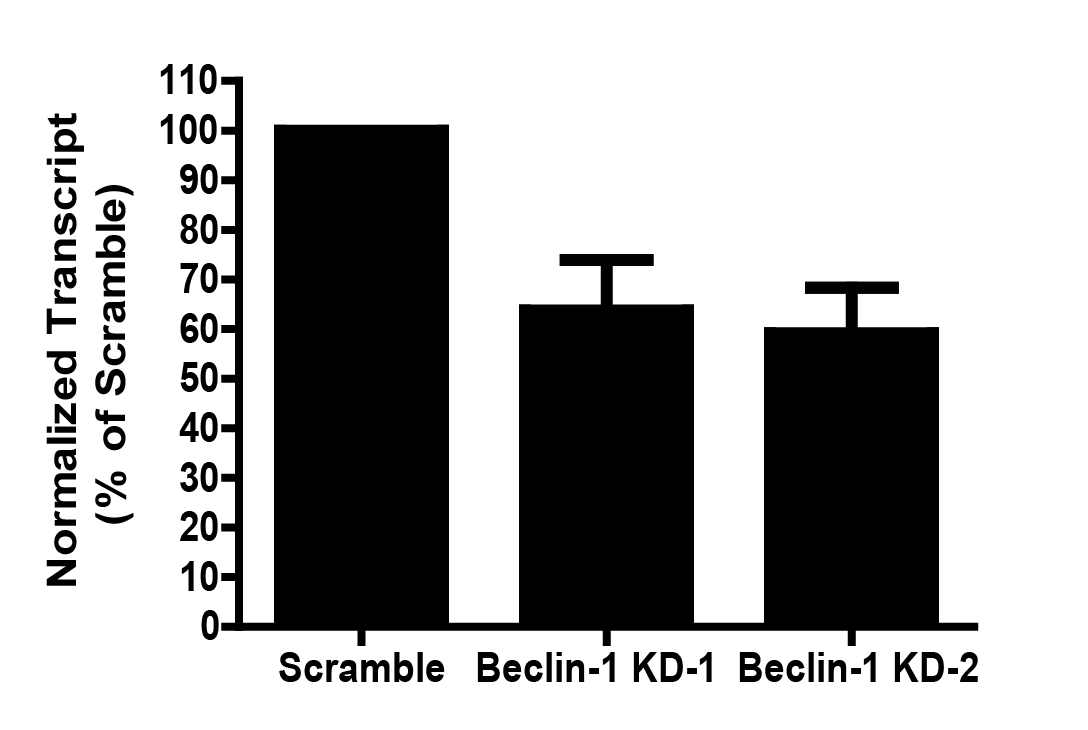

Supplement: Figure S4 — Beclin-1 shRNA depletes Beclin-1 mRNA in MEFs. qRT-PCR quantification of Beclin-1 mRNA in MEFs transduced with a lentivirus encoding a Beclin-1 or scramble shRNA. KD-1 and KD-2 are independently derived lines transduced with different Beclin-1 shRNA's. Results were normalized to GAPDH and are expressed as percent of the scramble control. (TIF) [file ppat.1003562.s004.tif]

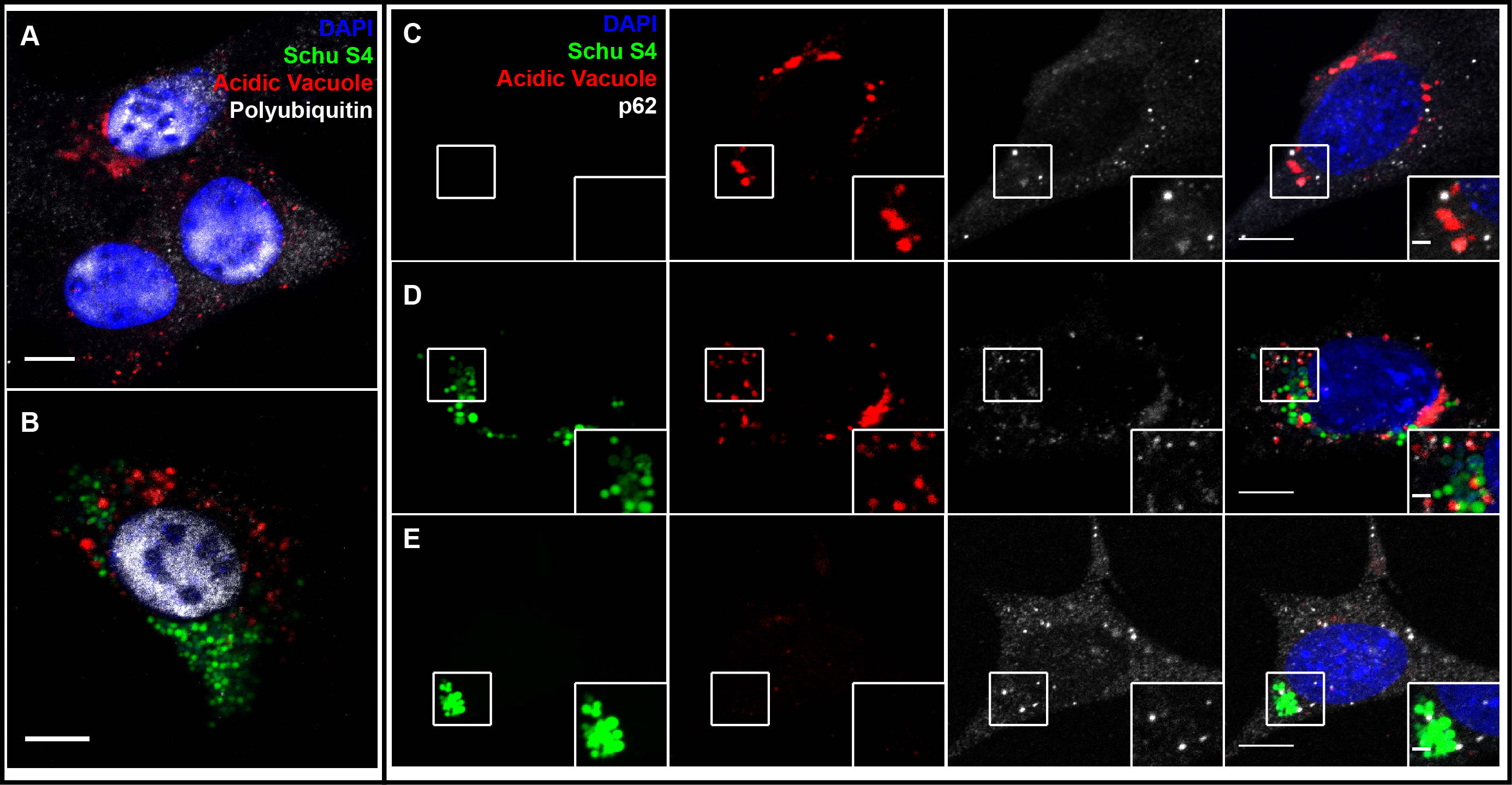

Supplement: Figure S5 — F. tularensis infection decreases polyubiquitin puncta but increases the number of p62+ acidic vacuoles. Representative fluorescence confocal microscopy images of (A) uninfected and (B) infected wild type MEFs depicting polyubiquitin. Representative fluorescence confocal microscopy images of (C) uninfected, (D) infected, or (E) infected 3MA treated wild type MEFs stained for p62/SQSTM1. Scale bars represent 10 µm at the low magnification and 2 µm for the higher magnification inset. Nuclei (DAPI) is depicted in blue, GFP-Schu is depicted in green, acidic vacuoles are depicted in red, and polyubiquitin or p62/SQSTM1 are depicted in white. (TIF) [file ppat.1003562.s005.tif]

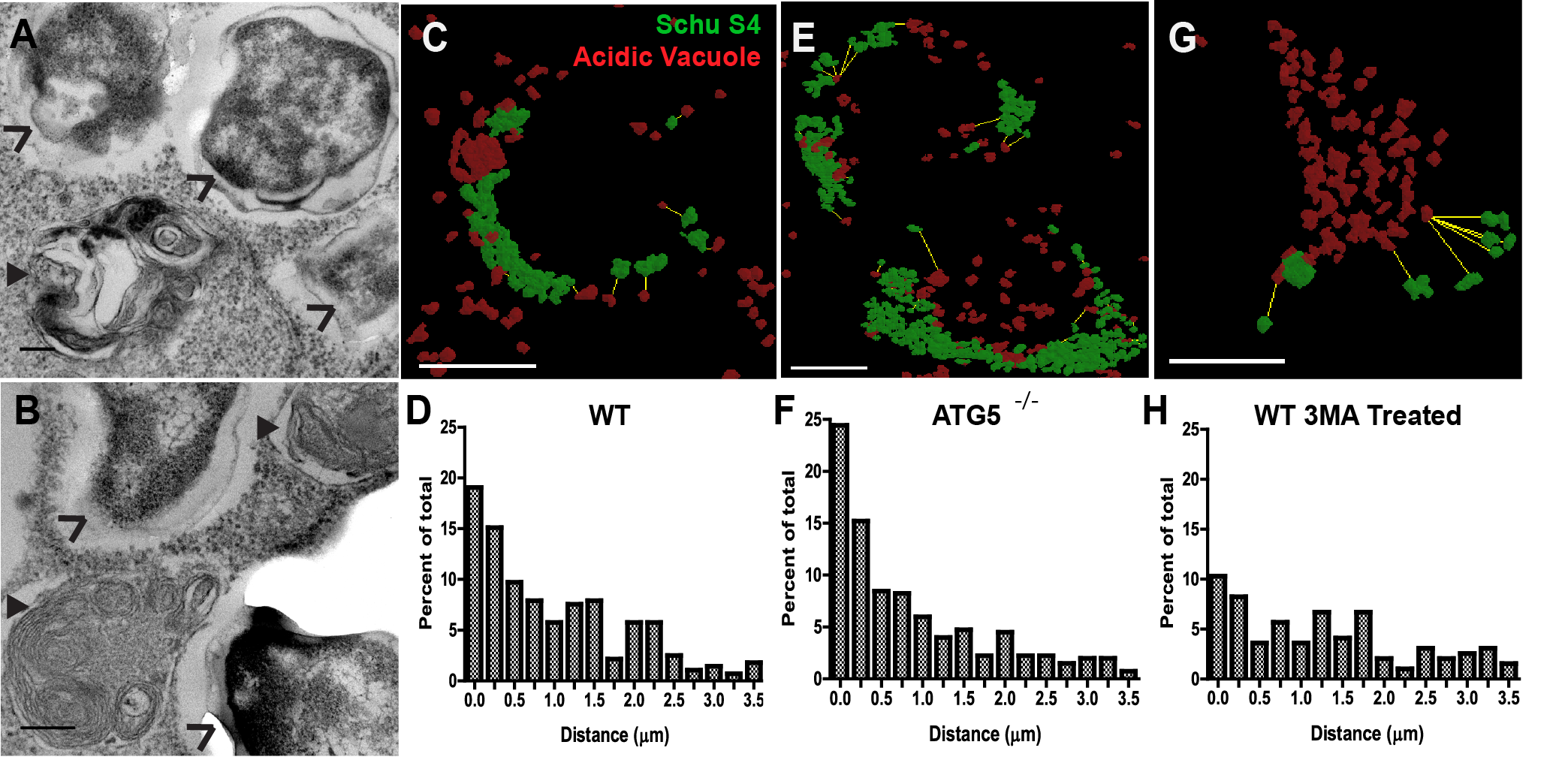

Supplement: Figure S6 — F. tularensis localizes adjacent to autolysosomes. Representative transmission electron (TEM) microscopy images of Schu S4 (open faced arrows [>]) adjacent to an autophagosome (solid arrows [▸]) in (A) J774 cells or (B) ATG5−/− MEFs 16 hours post inoculation. The scale bar for the TEM micrograph represents 200 nm. Representative compiled Z-stack images showing the distance (yellow line) between Schu S4 (green) and acidic vacuoles (red) in (C) wild type untreated, (E) ATG5−/− untreated or (G) wild type 3MA treated MEFs 16 hours post inoculation. Scale bars for the 3D images represent 10 µm. The distance between Schu S4 and the closest acidic vacuole in (D) untreated wild type (n = 342 bacteria), (F) ATG5−/− (n = 401 bacteria) or (H) 3MA treated wild type (n = 194 bacteria) MEFs. The distribution histograms are pooled from 3 independent experiments. (TIF) [file ppat.1003562.s006.tif]
