# Supplementary material for: Francisella tularensis Harvests Nutrients Derived via ATG5-Independent Autophagy to Support Intracellular Growth
Source: PLoS Pathog. 2013 Aug 15;9(8):e1003562. doi: 10.1371/journal.ppat.1003562 (PMC3744417; doi:10.1371/journal.ppat.1003562)
Supplement: Table S1 — Quantitative RT-PCR primer sequences. Primer sequences for assaying the amount of Beclin-1 or GAPDH mRNA in lentiviral transduced MEFs by qRT-PCR. (DOCX) [file ppat.1003562.s007.docx]

| **Primer** | **Sequence (5’ to 3’)** |
| --- | --- |
| Beclin-1 Forward | CAGCCTCTGAAACTGGACACGA |
| Beclin-1 Reverse | CTCTCCTGAGTTAGCCTCTTCC |
| GAPDH Forward | CATCACTGCCACCCAGAAGACTG |
| GAPDH Reverse | ATGCCAGTGAGCTTCCCGTTCAG |
